# Transient O_2_ pulses direct Fe crystallinity and Fe(III)-reducer gene expression within a soil microbiome

**DOI:** 10.1186/s40168-018-0574-5

**Published:** 2018-10-23

**Authors:** Jared Lee Wilmoth, Mary Ann Moran, Aaron Thompson

**Affiliations:** 1 Department of Crop and Soil Sciences, University of Georgia, Athens, 30602 GA USA; 2 Department of Marine Sciences, University of Georgia, Athens, GA USA

**Keywords:** Soil microbiome, Redox cycling, Microbial Fe(III) reduction, C cycling, Metatranscriptomics, Mössbauer spectroscopy

## Abstract

**Background:**

Many environments contain redox transition zones, where transient oxygenation events can modulate anaerobic reactions that influence the cycling of iron (Fe) and carbon (C) on a global scale. In predominantly anoxic soils, this biogeochemical cycling depends on Fe mineral composition and the activity of mixed Fe(III)-reducer populations that may be altered by periodic pulses of molecular oxygen (O_2_).

**Methods:**

We repeatedly exposed anoxic (4% H_2_:96% N_2_) suspensions of soil from the Luquillo Critical Zone Observatory to 1.05 × 10^2^, 1.05 × 10^3^, and 1.05 × 10^4^ mmol O_2_ kg^−1^ soil h^−1^ during pulsed oxygenation treatments. Metatranscriptomic analysis and ^57^Fe Mössbauer spectroscopy were used to investigate changes in Fe(III)-reducer gene expression and Fe(III) crystallinity, respectively.

**Results:**

Slow oxygenation resulted in soil Fe-(oxyhydr)oxides of higher crystallinity (38.1 ± 1.1% of total Fe) compared to fast oxygenation (30.6 ± 1.5%, *P* < 0.001). Transcripts binning to the genomes of Fe(III)-reducers *Anaeromyxobacter*, *Geobacter*, and *Pelosinus* indicated significant differences in extracellular electron transport (e.g., multiheme cytochrome *c*, multicopper oxidase, and type-IV pilin gene expression), adhesion/contact (e.g., S-layer, adhesin, and flagellin gene expression), and selective microbial competition (e.g., bacteriocin gene expression) between the slow and fast oxygenation treatments during microbial Fe(III) reduction. These data also suggest that diverse Fe(III)-reducer functions, including cytochrome-dependent extracellular electron transport, are associated with type-III fibronectin domains. Additionally, the metatranscriptomic data indicate that *Methanobacterium* was significantly more active in the reduction of CO_2_ to CH_4_ and in the expression of class(III) signal peptide/type-IV pilin genes following repeated fast oxygenation compared to slow oxygenation.

**Conclusions:**

This study demonstrates that specific Fe(III)-reduction mechanisms in mixed Fe(III)-reducer populations are uniquely sensitive to the rate of O_2_ influx, likely mediated by shifts in soil Fe(III)-(oxyhydr)oxide crystallinity. Overall, we provide evidence that transient oxygenation events play an important role in directing anaerobic pathways within soil microbiomes, which is expected to alter Fe and C cycling in redox-dynamic environments.

**Electronic supplementary material:**

The online version of this article (10.1186/s40168-018-0574-5) contains supplementary material, which is available to authorized users.

## Background

Soils in diverse terrestrial ecosystems (e.g., humid forests, wetlands, and irrigated agricultural lands) often undergo fluctuations in O_2_ during periodic wetting and drying events, and experience depletion of O_2_ during heterotrophic microbial respiration when the bulk soil or microsites within soil aggregates become saturated with water [1–4]. In soils, Fe(III) is typically the most abundant terminal electron acceptor for microbial respiration after O_2_. The soil microbiome is therefore strongly influenced by Fe during periods of low O_2_ because the microbial reduction of Fe(III) to Fe(II) can dominate important biogeochemical reactions [5–8]. Much of the Fe in soil exists as Fe(III)-(oxyhydr)oxides [9], but also as substituted Fe(III)/Fe(II) within clay minerals and as organo-Fe complexes [10–12]. Under both low and O_2_-rich conditions, Fe(III) phases are major sorbents for nutrients and organic matter and influence the flocculation and dispersion of soil colloids [13, 14]. The most disordered, lowest crystallinity Fe(III)-(oxyhydr)oxides (known as short-range-ordered (SRO) Fe phases) are widely understood to govern the abundance of organic C (OC) (along with SRO Al phases) in soils and sediments [15], especially in humid climatic regimes [16, 17]. During low-O_2_ conditions, microbial respiration on SRO Fe(III) phases typically results in their mineral dissolution and the loss of their OC- and nutrient-binding capability [18–20].

Pulsing of O_2_ or a return to O_2_-rich conditions following microbial Fe(III) reduction to Fe(II) promotes the re-precipitation of SRO Fe(III) phases, thereby renewing Fe(III) as an available electron acceptor for microbial respiration should low-O_2_ conditions return [5]. Recently precipitated Fe(III) mineral phases are often less crystalline and thus more reactive toward nutrient/carbon sorption and microbial reduction [9, 11, 21, 22]. Consequently, redox-driven transformations of Fe-(oxyhydr)oxides can have a profound effect on the mineralization of OC in soil microbiomes and by extension alter ecosystem-level processes such as C turnover rates, greenhouse gas emissions, and plant nutrient availability [23–31]. However, the underlying mechanisms of how different microbial Fe(III) reducers respond to the geochemical Fe species formed in redox-fluctuating systems remain poorly understood [5, 32–34].

The particle size and reactivity of Fe(III) minerals are influenced by the degree of disorder in the crystal structure, such that more disordered phases are typically smaller and have higher reactivity per mass, and are thus more energetically favorable as electron acceptors during microbial Fe(III) reduction [19–21, 35, 36]. Extracellular reduction of solid-phase Fe-(oxyhydr)oxides by Fe(III)-reducing bacteria (e.g., *Geobacter* and *Shewanella*) is facilitated by multiheme cytochromes, electrically conductive pili, flagella, biofilms, and soluble electron-shuttling compounds that can be regulated by differences in Fe crystallinity and reactivity [32, 33, 37–44]. Compared to pure culture experiments, rapid oxygenation of anoxic soil suspensions containing native Fe minerals and mixed Fe(III)-reducer populations has been shown to regulate microbial Fe(III) reduction rates during subsequent anoxic conditions, likely through the production of more available/reactive Fe(III) phases relative to bulk soil Fe [6, 7]. Consequently, the transcriptomic activity of mixed Fe(III)-reducer populations in the soil microbiome should reflect important changes in Fe and C cycling [28, 33, 45, 46].

Unlike synthetic Fe(III) phases often used in relevant gene expression studies [32, 33, 39, 43], Fe minerals found in soil are often highly substituted with foreign ions or organic compounds and have disordered crystal structure [47–51]. During the laboratory synthesis of Fe(III)-(oxyhydr)oxides, slower or faster rates of Fe(II) oxidation by O_2_ lead to the formation of either more crystalline or less crystalline Fe(III) phases, respectively [52–55]. Additionally, many researchers have found that when Fe(III) phases are exposed to aqueous or sorbed Fe(II), which occurs during microbial Fe(III) reduction, mineral ripening (Ostwald) increases along with changes in Fe(III) crystallinity [36, 56]. The crystallinity of soil Fe(III) minerals can increase [27, 57, 58], decrease [22, 59], or remain unchanged/undetected [34] in response to redox fluctuations; however, the mechanisms governing these processes are still unclear [6, 57, 60].

To examine the effect of transient oxygenation on Fe(III)-reducer gene expression and Fe mineral transformations in soil, we repeatedly exposed the headspace of anoxic soil suspensions to pulses of O_2_ at different influx rates. We chose soil from the Bisley Watershed of the Luquillo Critical Zone Observatory (LCZO), Puerto Rico, because it has a well-documented history of redox fluctuations in the field and is well characterized in terms of mineral and microbial composition [2, 6, 18, 61]. We tested the hypothesis that slow oxygenation rates lead to the buildup of more crystalline Fe(III) phases and characterized how Fe(III)-reducer gene expression is affected under different treatment conditions. Variable temperature ^57^Fe Mössbauer spectroscopy was used to track changes in Fe mineral composition while metatranscriptomic analysis was used to measure the presence and relative abundance of mRNA transcripts binning to Fe(III)-reducer genomes. Our study demonstrates that combining ^57^Fe Mössbauer spectroscopy and a metatranscriptomic analysis of Fe(III)-reducing taxa provides a novel view of the mineral-microbe interface in redox-fluctuating systems.

## Methods

### Sample collection

Soil was collected from the Bisley Watershed (LCZO), Luquillo Experimental Forest, Puerto Rico. Soil characteristics, geographic coordinates, and site-specific details including annual precipitation have been reported elsewhere [2, 6, 18, 62]. Sample processing details are given in Additional file 1: Section 1.

### O_2_ pulse methodology

We used 120 ml dark amber serum bottles (Wheaton, Millville, NJ, USA) as microcosms during a 31-day anoxic incubation with three 7-h pulses of O_2_ at different delivery rates initiated every 7 days. Soils (2 g, air dried) were placed in serum bottles and moved into an anoxic chamber (Coy Labs, Grass Lake, MI, USA; 4% H_2_:96% N_2_), mixed with 20 ml of anoxic 25 mM (2-(*N*-morpholino)ethanesulfonic acid (MES) + KCl) buffer (pH 6), sealed with gray butyl stoppers and aluminum crimp caps, and then mounted on a rotary shaker in the anoxic chamber. To induce oxygenation following 7-day anoxic conditions, [O_2_]_atm_ (21% O_2_ in air) was added to triplicate microcosms over an initial 7 h period (i.e., at the onset of oxygenation), each hour, as 2.1 × 10^−4^ mol h^−1^ (slow treatment), 10^−3^ mol h^−1^ (medium treatment), or 10^−2^ mol h^−1^ (fast treatment). Following 7 h of oxygenation, an additional 17 h was allowed to pass before again exposing the microcosms to an anoxic headspace in the chamber (i.e., total oxic time = 24 h). A single addition to the slow, medium, and fast oxygenation treatments introduced 1.05 × 10^2^, 1.05 × 10^3^, and 1.05 × 10^4^ mmol O_2_ kg^−1^ soil, respectively, and would theoretically be able to oxidize 4.2 × 10^2^, 1.05 × 10^3^, and 1.05 × 10^4^ mmol Fe(II) kg^−1^ soil at pH 6, respectively, if Fe(II) was the only species oxidized [52]. This cycle of 7 days of anoxic conditions and a 7-h pulse of O_2_ was repeated three times and then followed finally by 7 days of anoxic conditions to complete the 31-day incubation (see Additional file 1: Section 1 for more details).

### Incubation sampling scheme and Fe chemical analyses

Samples (1 ml) were collected for Fe(II) chemical analysis using sterile 10-ml plastic syringes fitted with wide bore needles (16 ga; 1.2 mm id; sterile stainless steel) at 0 day, at the end of each 7-day anoxic period, and after 1, 3, and 24 h during each oxic period unless otherwise indicated (see Additional file 1: Section 1). Aliquots were centrifuged at 21,000 rcf for 15 min and returned to the anoxic chamber for collection of the aqueous phase, and then, the remaining soil pellet was extracted with 0.5 M HCl for 2 h in the dark and centrifuged at 21,000 rcf for 15 min prior to collection of the acid supernatant. Measurement of aqueous and acid extractable Fe(II) was carried out using a modified ferrozine assay [57], and Fe_Total_ in the initial soil was determined using lithium-metaborate fusion (sub-contracted to Australian Laboratory Services (ALS) Minerals, Reno, NV, USA).

### Variable temperature ^57^Fe Mössbauer spectroscopy

Samples collected under oxic conditions for Mössbauer spectroscopy at 24 days during the incubation were centrifuged as described above for Fe chemical analyses, and the supernatants were subsequently removed in the anoxic chamber. Soil pellets remaining in the micro-centrifuge tubes were sealed under anoxic headspace and stored at − 80 °C until further analysis. Frozen samples were transferred to the anoxic chamber and prepared as sealed mounts for Mössbauer spectroscopy measurements. For each treatment, replicate soil gels (*n* = 3) were mixed (i.e., pooled) (180 mg per total equivalent dry mass mount) within the cavity of a thin nylon ring and sealed between two layers of Kapton tape. The mounts were then frozen and introduced to a He-cooled Mössbauer spectrometer at low temperature (< 100 K) to prevent changes in mineralogy.

Absorption spectra of the initial air-dried soil and sampled incubation soils were collected in transmission mode with a variable temperature He-cooled cryostat (Janis Research Co.) and a 1024 channel detector. A ^57^Co source (~ 50 mCi) embedded in a Rh matrix was used at room temperature. Velocity (i.e., gamma-ray energy) was calibrated using α-Fe foil at 295 K, and all center shift (CS) and peak positions were reported with respect to this standard. The transducer was operated in constant acceleration mode, and the spectra were folded to 512 channels to achieve a flat background. Mössbauer spectral fitting was performed using Recoil™ software (ISA Inc.) with the Voigt-based fitting (VBF) method. The Lorentzian linewidth (HWHM) parameter was held at 0.1425 mm s^−1^, corresponding to the measured linewidth using α-Fe foil at 295 K on our instrument. Mössbauer spectroscopy methods and associated references are given in detail in Additional file 1: Section 2.

### RNA isolation, sequencing, and analysis

Methods for RNA extraction and sequencing followed Gifford et al. [63] and Tveit et al. [64]. Soil suspensions collected for RNA sequencing at 31 days were transferred inside the anoxic chamber and then immediately frozen in liquid N_2_ and subsequently stored at − 80 °C until further analysis. Samples were prepared for RNA extraction by transferring frozen fragments to bead tubes (Mo Bio, Carlsbad, CA, USA) on dry ice. A stream of N_2_ was used to flush the headspace of each bead tube and then capped to prevent any dramatic redox changes in the steps that followed. To begin extraction (1 g per dry soil mass equivalent extraction), the sealed N_2_-flushed tubes were quickly moved between ice and brief vortexing to initiate thawing. The samples were then immediately placed in a 0 °C centrifuge and spun at 4,000× rcf for 7 min. Removal of the separated aqueous phase after centrifugation marked the immediate transition to step 1 of total RNA extraction as detailed in the RNA PowerSoil® Total RNA Isolation Kit (Mo Bio) user protocol, and total RNA was then extracted from soils following the manufacturer’s instructions. All mass transfers that occurred during extraction were recorded during the procedure to calculate RNA yields on a dry soil basis. Initial RNA yields and purity were measured on a NanoDrop spectrophotometer.

The Bacteria Ribo-Zero Magnetic Kit (Epicenter-Illumina, Madison, WI, USA) was used to remove ribosomal RNA (rRNA). Linear amplification of the resuspended rRNA-depleted samples was performed using the MessageAmp II-Bacteria Kit (Ambion®, Thermo Fisher Scientific, Waltham, MA, USA). Fragment size analysis and integrity of the mRNA-amplified samples were checked on a TapeStation 2200 (Agilent Technologies, Santa Clara, CA, USA). Sequence cDNA libraries were constructed, following quality control assessment on a BioAnalyzer 2100 (Agilent), using the KAPA Stranded RNA-Seq kit (Kapa Biosystems, Wilmington, MA, USA) with TruSeq adapters (Illumina, San Diego, CA, USA). Libraries were pooled and sequenced on four lanes using the NextSeq platform (Illumina) to generate 150 nt paired-end sequences. Library construction, process quality control, and sequencing were performed at the Georgia Genomics and Bioinformatics Core, Athens, GA, USA.

Sequence quality was evaluated using the program FastQC. PEAR was used to merge paired-end sequences, and Prinseq was used to trim poly-A/T tails ≥ 15 nt added in the RNA amplification procedure. RiboPicker was used to remove rRNA sequences (16S, 23S, 18S, 28S, 5S, and 5.8S units) using a standalone non-redundant rRNA database (rrnadb) which included the most current versions of SILVA, Ribosomal Database Project RDP-II, GreenGenes, NCBI archeal/bacterial complete genomes rRNA and Rfam databases. Alignments of the remaining sequences were performed with DIAMOND using sensitive-*BLASTX* searches (bit score ≥ 40, *E* value ≤ 10^−3^) against the NCBI RefSeq protein database. MEGAN6 software was used to bin putative mRNA sequences taxonomically (RefSeq) and functionally (Interpro) and was also used to perform principal coordinates analysis (PCoA). Distance matrices for PCoA analysis were calculated using Bray-Curtis distances in MEGAN6. Values for the number of sequences surviving each step of processing are given in Additional file 2: Table S1. Significant differences in relative gene expression between treatments were measured using DESeq2. Statistical values are those reported by DESeq2 using an adjusted *P* cutoff of 0.1. A complete RefSeq accession-to-annotation key is given in Additional file 3: Table S2. The primary data handling and analyses were performed on a Linux cluster at the Georgia Advanced Computing Resource Center, Athens, GA, USA.

All original sequence data are under BioProject PRJNA381321 in the NCBI SRA database (https://www.ncbi.nlm.nih.gov/Traces/study/?acc=SRP103306). The corresponding sample accessions are listed in Additional file 2: Table S1. A full account of the above RNA sampling and analysis pipeline, including the associated references, is included in Additional file 1: Section 1.

## Results

### Fe(II) chemical analysis

**Fig. 1:**
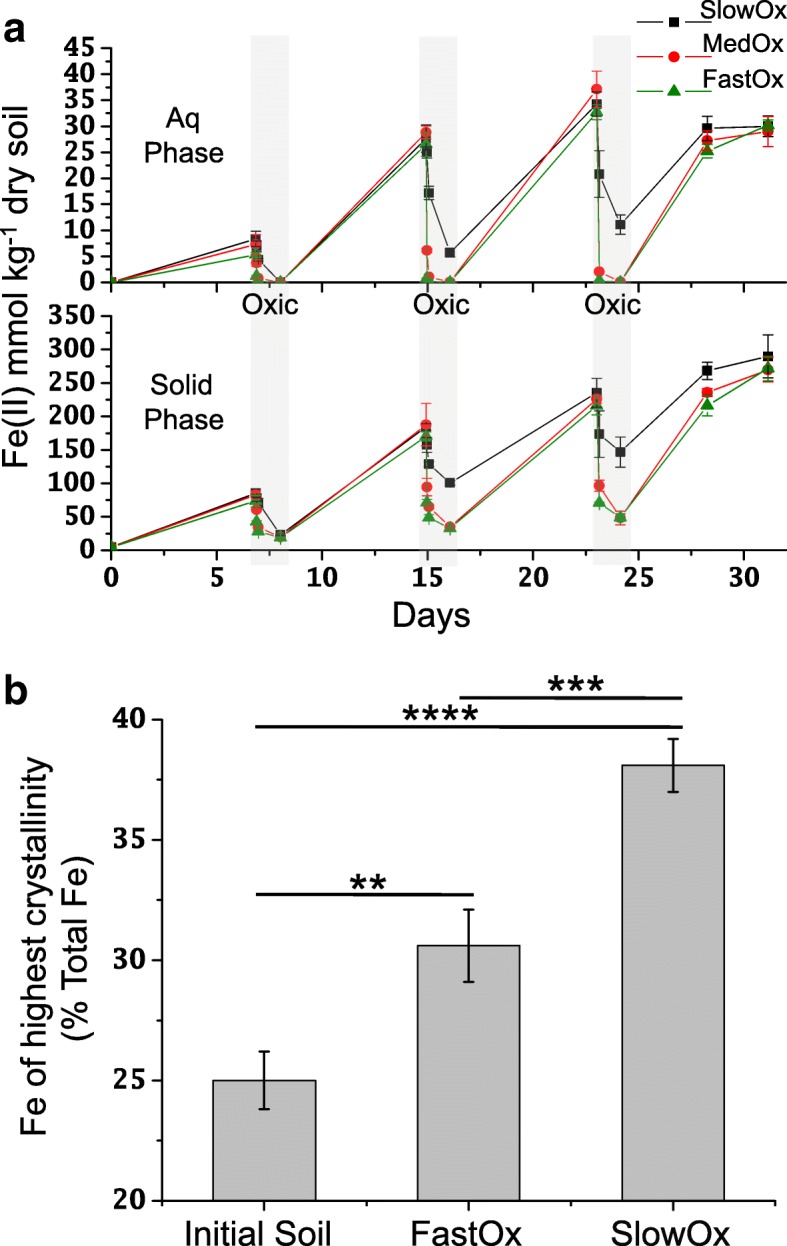
Fe(II) concentrations and Fe(III) solid phase crystallinity under different oxygenation treatments. **a** Amount of Fe(II) extracted from the aqueous and solid phases when [O_2_]_atm_ (21% O_2_ in air) was supplied at 2.1 × 10^−4^ (SlowOx), 2.1 × 10^−3^ (MedOx), or 2.1 × 10^−2^ (FastOx) mol h^−1^. Data points are means (± s.d.) (*n* = 3). Light gray vertical bars denote 24-h periods under oxic conditions, see Additional file 1: Figure S1 for expanded view of oxic intervals. **b** Mössbauer results showing differences in Fe crystallinity between oxygenation treatments. *T* test significance: **P < 0.01, ***P < 0.001, ****P < 0.0001. Triplicate samples were pooled prior to Mössbauer analysis. The error bars represent standard deviations in the signal modeling (see Additional file 1: Section 2 for details)

Anoxic Fe(II) concentrations were similar at the end of the experiment for all treatments in both the aqueous and solid phases (Fig. 1a) and increased as the incubation progressed, consistent with prior work which used air-dried soils from this site [6, 7]. HCl-extractable Fe(II) approached 300 mmol kg^−1^ by 31 days in all treatments, whereas Fe(II)_aq_ concentrations never increased beyond *ca* 35 mmol kg^−1^, consistent with the Fe(II) sorption isotherm for this soil (Additional file 1: Figure S13). Following oxygenation at both 16 and 24 days (i.e., the end of redox cycles two and three), aqueous and HCl-extractable Fe(II) remained higher in the slow treatment (Fig. 1a). By the end of the incubation, Fe(II) concentrations in all treatments reached 29% of Fe_Total_ (Fe_Total_ = 1162.5 ± 17.5 mmol kg^−1^ soil). There was no significant difference in Fe(III) reduction rates (aq + solid Fe(II)) between treatments over the last 3 days of the incubation.

### Mössbauer analysis of soil Fe phase crystallinity

Soils at the initiation of the experiment, those collected at 24 days following fast oxygenation, and those at 24 days following slow oxygenation all had prominent sextet-components in the 77 Kelvin (K) Mössbauer spectra that accounted for 25 ± 1.2, 30.6 ± 1.5, and 38.1 ± 1.1% Fe_Total_, respectively, which indicates increasing Fe(III)-(oxyhydr)oxide crystallinity (initial < fast treatment < slow treatment) (Fig. 1b). There was a significantly larger sextet-component contribution in the slow oxygenation treatment compared to the fast oxygenation treatment (*T* test; *P* < 0.001), and in the fast oxygenation treatment compared to the initial soil (*P* < 0.01). In all cases, the sextets had spectral parameters most closely associated with nano-goethite (α-FeOOH) (see Additional file 1: Section 2 and Additional files 4, 5, 6, and 7). For the initial soil and treatments, we found that at least 50% of Fe(II) displayed weak magnetic order at 4.5 K. We did not include the medium oxygenation treatment in our final Mössbauer or metatranscriptomic analyses (see also Additional file 1: Section 1); the following sections report only the metatranscriptomic data from the fast and slow oxygenation treatments.

### Microbial community attributes

**Fig. 2:**
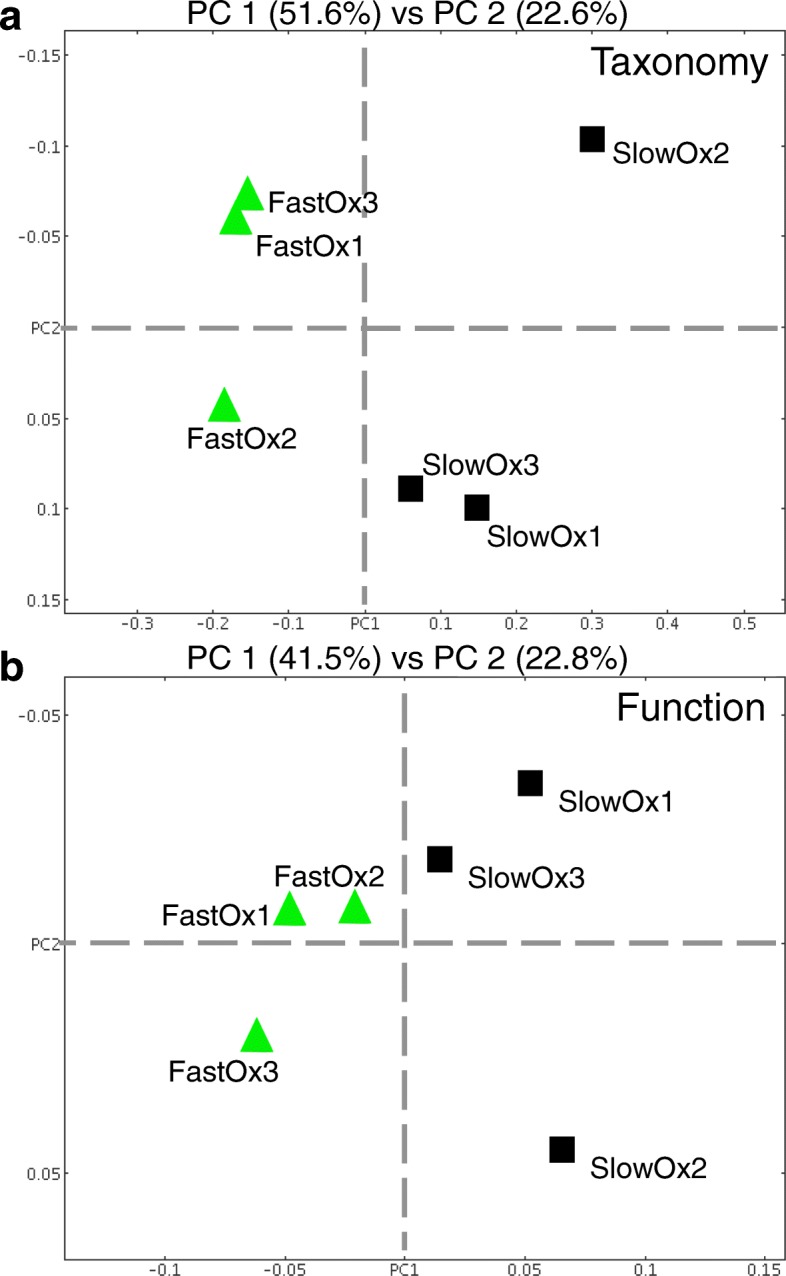
Principal coordinates analysis (PCoA) for oxygenation treatments using Bray-Curtis distances. **a** Genus assignments in MEGAN6 using NCBI taxonomy classifications. **b** Functional assignments in MEGAN6 using Interpro classifications

Microbial taxa were identified based on transcripts recruiting to reference genomes at both the genus and species level following normalization of total transcript counts per sample in MEGAN6. The number of active species detected in the fast oxygenation treatment (1020 ± 80) was significantly greater than in the slow oxygenation treatment (870 ± 40) (*T* test; *P* < 0.05; Additional file 1: Figure S2). The PCoA results based on taxonomic and functional assignment of transcripts show that replicate samples exposed to the same oxygenation treatment tended to group together (Fig. 2).

### Important Fe(III) reducers

Overall, 543 ± 59 and 638 ± 54 genera were detected in the slow and fast oxygenation treatments, respectively. Rankings of the top 50 genera by relative contribution to the metatranscriptome indicate that bacteria belonging to the genera *Anaeromyxobacter*, *Geobacter*, and *Pelosinus* were major Fe(III) reducers in both treatments (Additional file 1: Figure S3). The relatively large metatranscriptomic contribution of these three genera is consistent with the geochemical profile of the humid forest soil used in our experiment, which is replete with nano-phase Fe-(oxyhydr)oxides. Specifically, *Anaeromyxobacter* and *Geobacter* are known to express characteristic genes during extracellular electron transfer to Fe(III), such as multiheme cytochrome *c* genes, which is expected to depend on Fe mineral crystallinity/reactivity in soils [32, 33, 65]. Recent investigations have identified *Pelosinus* as a predominant Fe(III)-reducing genus in diverse anaerobic and heavy-metal rich environments, where specific extracellular matrix components of *Pelosinus* (e.g., S-layer complexes) mediate reactions with heavy metals [66–69]. Other minor and less well-known microbial contributors to extracellular Fe(III) reduction were likely present, but our metatranscriptomic focus on transcripts recruiting to *Anaeromyxobacter*, *Geobacter*, and *Pelosinus* genomes provided a more solid biological basis for our analysis of characteristic Fe(III)-reducer activity within the soil microbiome.

### Differentially expressed and other essential genes for Fe(III) reduction following soil oxygenation at different rates

**Fig. 3:**
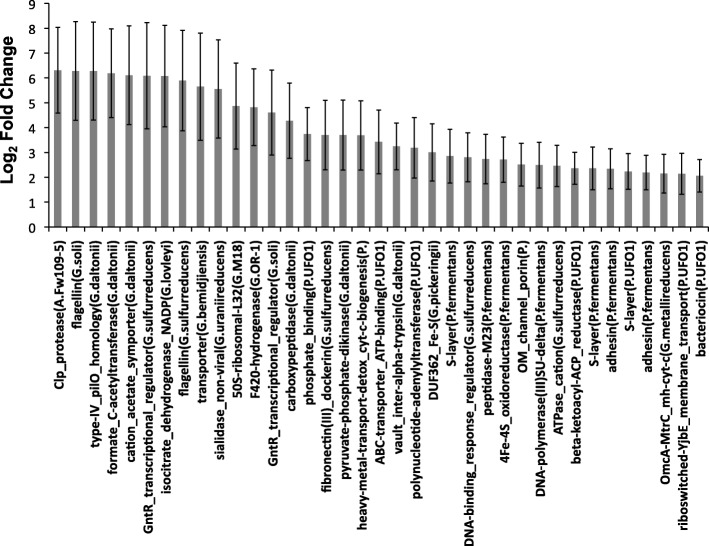
All differentially expressed genes of *Anaeromyxobacter*, *Geobacter*, and *Pelosinus* detected at significantly higher levels in the fast oxygenation treatment (DESeq; *P* < 0.01). Bars indicate means (± s.e.) (*n* = 3). Taxonomic annotation is shown in parentheses denoted by A. (*Anaeromyxobacter*), G. (*Geobacter*), or P. (*Pelosinus*). A complete RefSeq accession-to-annotation key is given in Additional file 3: Table S2

**Fig. 4:**
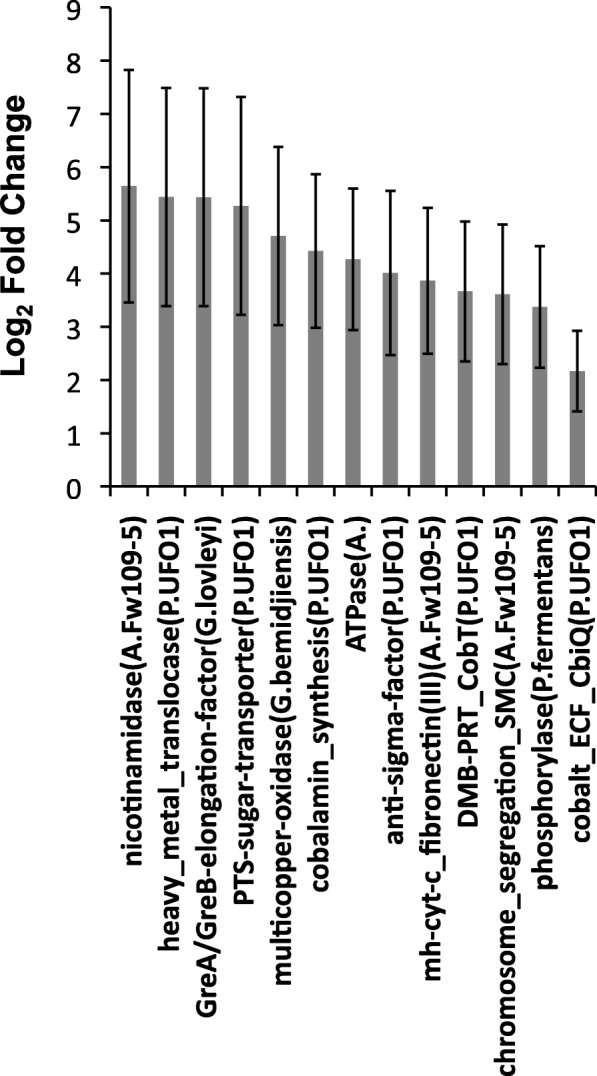
All differentially expressed genes of *Anaeromyxobacter*, *Geobacter*, and *Pelosinus* detected at significantly higher levels in the slow oxygenation treatment (DESeq; *P* < 0.01). Bars indicate means (± s.e.) (*n* = 3). Taxonomic annotation is shown in parentheses denoted by A. (*Anaeromyxobacter*), G. (*Geobacter*), or P. (*Pelosinus*). A complete RefSeq accession-to-annotation key is given in Additional file 3: Table S2

We measured significantly higher relative expression for an *omcA*/*mtrC* (outer membrane) multiheme cytochrome *c* gene, a type-IV pilin *pilO*-homology gene, and flagellin genes in the fast oxygenation treatment than in the slow treatment, consistent with changes in extracellular Fe(III) reduction (Fig. 3). Membrane adhesin and S-layer genes, which we believe to be involved in the reduction of Fe(III), and a vault protein inter-alpha-trypsin (*VIT*) gene also showed significantly higher relative expression in the fast treatment. *VIT* proteins form nano-cage structures that are important for transport and extracellular matrix stabilization (e.g., biofilms) [70], which are expected to be essential processes during microbial Fe(III) reduction in soil microbiomes. A gene containing domains for both heavy metal transport and cytochrome *c* biogenesis and a 4Fe-4S oxidoreductase gene also showed significantly higher relative expression in the fast treatment than in the slow treatment that is consistent with Fe(III) reduction. Also showing significantly higher expression in the fast treatment were fibronectin and sialidase genes that in the closest reference genomes contain cellulosome-associated type-I dockerin domains, suggesting involvement in the degradation of cellulose. This indicates that cellulose might serve as an important organic C (OC) electron donor for Fe(III) reduction in soils. There were fewer genes that showed significantly higher relative expression in the slow oxygenation treatment compared to the fast oxygenation treatment (Fig. 4). These included a multicopper oxidase gene, a multiheme cytochrome *c*/fibronectin type-III domain gene, and a heavy metal translocase gene that are all consistent with changes in extracellular Fe(III) reduction.

**Fig. 5:**
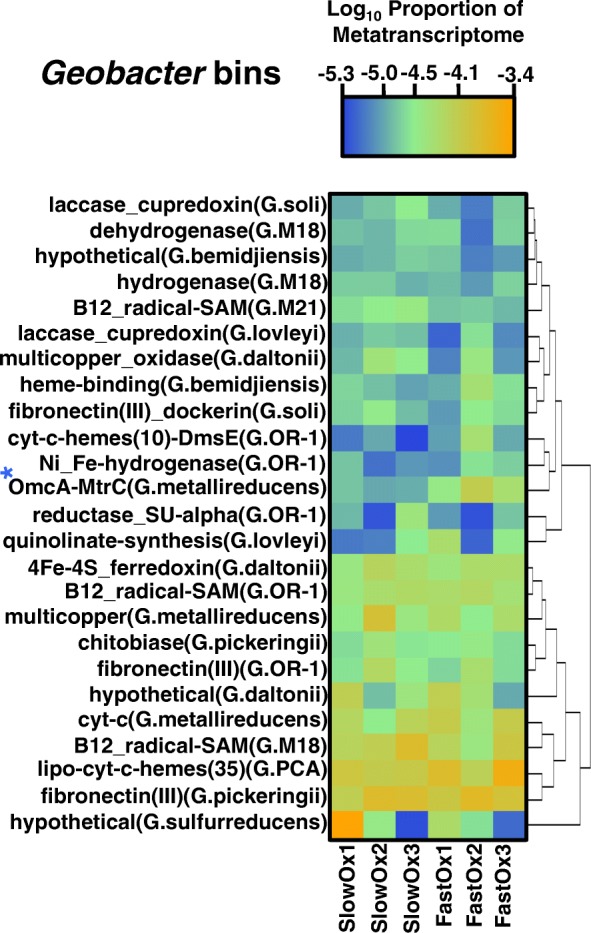
Heat map of the top ranking transcripts binning to *Geobacter* genomes based on relative expression. Tile color gradients represent log_10_ values of the metatranscriptome proportion in each treatment. Dendrograms were constructed using Euclidean distances. Blue asterisks indicate genes that were also differentially expressed (DESeq; *P* < 0.01) as shown in Fig. 3. A complete RefSeq accession-to-annotation key is in Additional file 3: Table S2

**Fig. 6:**
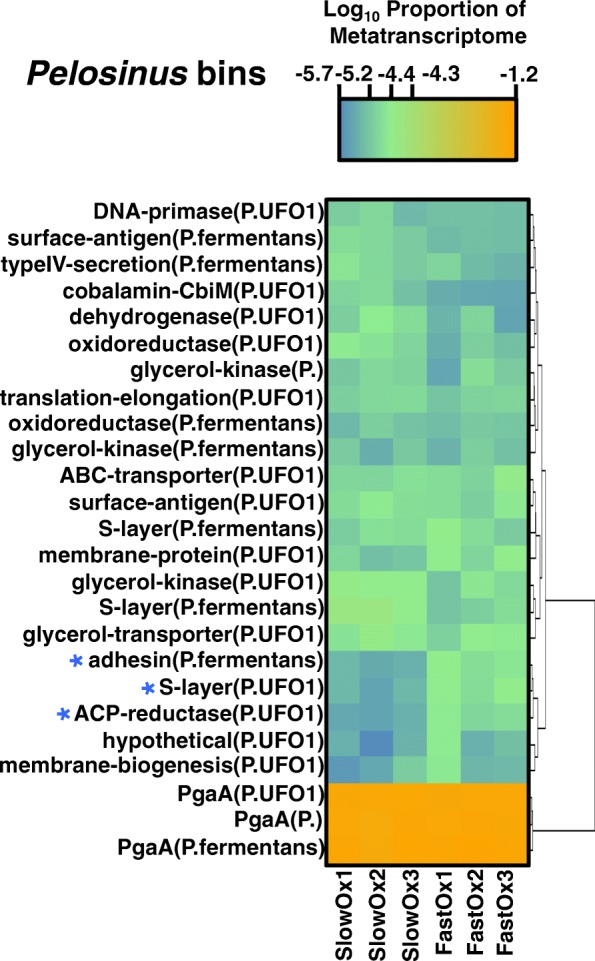
Heat map of the top-ranking transcripts binning to *Pelosinus* genomes based on relative expression. Tile color gradients represent log_10_ values of the metatranscriptome proportion in each treatment. Dendrograms were constructed using Euclidean distances. Blue asterisks indicate genes that were also differentially expressed (DESeq; *P* < 0.01) as shown in Fig. 3. A complete RefSeq accession-to-annotation key is in Additional file 3: Table S2

Other expressed genes that are informative about Fe cycling, but that were not significantly different in relative expression between the fast and slow treatment, match multicopper oxidase, fibronectin type-III domain, cytochrome *c*, multiheme cytochrome *c*, and 4Fe-4S genes of *Geobacter*, including genes likely involved in C and H_2_ metabolism (i.e., encoding dehydrogenase and hydrogenase enzymes) (Fig. 5). Similar genes required for anaerobic Fe(III) reduction were previously reported to be more highly expressed in uranium (U)-contaminated subsurface sediments compared to defined growth media [46]. Fibronectin type-III domain genes matching to *Geobacter* that also encode dockerin, cupredoxin, and laccase enzymes were highly expressed in our study. Importantly, these enzymes may be used for insoluble Fe(III) reduction [42], perhaps owing to the redox activity of multicopper constituents and/or the ability to oxidize organic compounds during electron transfer from soil OC to Fe(III). A highly expressed chitobiase gene matching to the *G*. *pickeringii* genome may suggest involvement in chitin degradation and consequently electron donation from this type of recalcitrant OC during Fe(III) reduction in the soil. The transcripts recruiting to *Pelosinus* genomes with high relative expression in both treatments included different S-layer encoding genes (Fig. 6), which we expect to play a vital role in the assembly and reduction of Fe(III) in the soil. However, the expression of certain S-layer genes is likely more characteristic for changes in Fe(III)-(oxyhydr)oxide crystal order as indicated in Fig. 3. The three top-most expressed genes of *Pelosinus* (Fig. 6) in both treatments are related to *pgaA* (poly-beta-1,6 *N*-acetyl-d-glucosamine export porin) of *Cupriavidus basilensis*. The *pgaA* gene is necessary for biofilm formation in Gram-negative bacteria, and based on our findings, could be involved in the cellular contact/reduction of reactive Fe(III)-(oxyhydr)oxides by *Pelosinus* in the soil.

## Discussion

This study examined Fe crystallinity transformations and Fe(III)-reducer gene expression in anoxic humid-forest soil microcosms repeatedly exposed to pulsed oxygenation at either a fast or slow rate. This experimental methodology served as an analog for redox-fluctuating conditions expected to occur in moist aggregates/microsites during wetting and drying of the bulk soil matrix.

### Fe crystallinity depends on oxygenation rates during redox oscillations

We tested the hypothesis that slow oxygenation rates lead to the buildup of more crystalline Fe(III) phases during soil redox oscillations. The Mössbauer data support this hypothesis, showing that slower oxygenation resulted in higher Fe(III)-(oxyhydr)oxide crystallinity than faster oxygenation (Fig. 1b and Additional file 1: Section 2). We expect that different Fe(II) oxidation rates strongly contributed to this result [52, 55, 71]. However, higher Fe(II) concentrations near the end of 16 and 24 day oxic intervals in the slow oxygenation treatment may have also promoted more crystal ripening (Ostwald) than in the fast treatment by facilitating greater abiotic electron transfer and re-crystallization [8, 58, 62]. This might also explain why both treatments showed significantly higher Fe crystallinity than the initial soil, since both treatments experienced elevated levels of Fe(II) during the incubation (Fig. 1a–b). Additionally, because the degree to which organic and inorganic substances are incorporated in de novo Fe(III) solids during precipitation is directly related to precipitation rate [72, 73], the fast treatment may have resulted in lower Fe crystallinity due to more precipitation of substituted Fe phases compared to in the slow treatment. Toward the end of incubation, Fe(II) in the solid phase began to plateau at *ca* 300 mmol kg^−1^ soil in both treatments (Fig. 1a), which is consistent with Fe(II) sorption (Additional file 1: Figure S13). This accumulated Fe(II) may have blocked some (oxyhydr)oxide surfaces during microbial Fe(III) reduction [19]. The weakly magnetic Fe(II) phase detected in all samples at 4.5 K is consistent with the sorption of biogenic Fe(II) to magnetically ordered Fe(III) phases, or possibly the presence/formation of magnetite [74] (Additional file 1: Figures S6–S8).

### Fe(III)-reducer gene expression in the soil microbiome reflects transformations in Fe crystallinity

Our data suggest that the strategies of Fe(III) reduction by native soil Fe(III) reducers are both sensitive to and indicative of relatively small differences in Fe(III) crystallinity induced by repeated O_2_ pulses at different rates. Subtle changes in the reactivity of various synthetic Fe minerals have also been shown to finely regulate Fe(III)-reduction pathways in pure culture experiments [32]. We observed several significant differences in relative gene expression between treatments that are consistent with microbial strategies for adapting to shifts in soil Fe mineral crystallinity [39, 42, 65]. One particular multiheme cytochrome *c* gene encoding a type-III fibronectin domain was expressed significantly higher in the slow treatment (Fig. 4), which suggests that fibronectin-like domains play a unique role in cytochrome-dependent extracellular electron transport in soil during increases in Fe mineral crystallinity. Overall, diverse Fe(III)-reducer functions were associated with type-III fibronectin domains in our experiment, which supports and broadens the results of earlier studies [42, 75]. The *omcA*/*mtrC* gene has been shown to be more essential for extracellular electron transfer during growth with Fe(III)-(oxyhydr)oxide compared to Fe(III)-citrate [39]; however, our data indicate that this gene is also sensitive to differences in soil Fe(III) crystallinity, being expressed significantly higher in the fast oxygenation treatment compared to slow oxygenation (Fig. 3). Flagellin genes and a type-IV *pilO*-homology gene in our study appeared to be more essential during reduction of less crystalline Fe(III) phases in the fast oxygenation treatment compared to the slow treatment (Fig. 3). Expression of flagellin genes was shown previously to increase ≥ 30 fold in *G. metallireducens* when grown on Fe(III)-(oxyhydr)oxide compared to Fe(III)-citrate [39] and are thought to be important for Fe mineral surface sensing and congregation at the mineral-microbe interface [33, 37, 40]. The expression of a *pilO*-like gene in our study was possibly involved in the formation of microbial nanowires that may be used to transfer electrons to mineral surfaces and/or other neighboring cells [76]. The significantly higher expression of flagellin and pilin genes in the fast oxygenation treatment also contrasts previous research [31] that showed significant downregulation of similar genes under O_2_ exposure and oxidative stress. Accordingly, the significantly higher expression of these genes observed in our fast oxygenation treatment under anoxic conditions supports our conclusion that flagellin and pilin gene expression reflected differences in Fe crystallinity during our experiment, and not oxidative stress.

The high metatranscriptomic contribution of transcripts recruiting to *Pelosinus* in both treatments (Additional file 1: Figure S3) may reflect the general availability of short range ordered (SRO) Fe phases in the soil [66, 77]. However, significantly higher relative expression of *Pelosinus* S-layer and adhesin genes (Fig. 3) is consistent with the more abundant SRO Fe(III) phases in the fast oxygenation treatment compared to the slow treatment. S-layer and adhesin proteins can be used by bacteria to transport mammalian heme to supply Fe during pathogenesis [78, 79]; therefore, it may be that Fe(III) reducers in soil can bind and transport Fe using similar mechanisms. Accordingly, Thorgersen et al. [68] has recently shown that large, highly expressed, S-layer complexes in *Pelosinus* UFO1 can bind heavy metals such as U(IV). The significantly higher relative expression of a heavy metal transport/cytochrome *c* biogenesis gene of *Pelosinus* in the fast oxygenation treatment also suggests a specific activity and function toward the reduction of more SRO Fe(III) phases, though it remains unclear if *Pelosinus* species can use cytochrome-mediated electron transfer to reduce Fe(III) [67]. Corrinoid (e.g., vitamin B_12_) genes matching to *Pelosinus* appear to have been more essential in the slow oxygenation treatment (Fig. 4), consistent with the higher relative expression of *cbiM* (which is involved in the uptake of Co atoms) of *Pelosinus* UFO1 in the slow treatment (Fig. 6). The enzymes encoded by *pdgle* and *cbiQ* are required for the transport of cobalt and the initial steps of corrinoid synthesis, while the enzyme encoded by *cobT* is required for catalyzing attachment of precursor components of vitamin B_12_ [80, 81]. Fluctuation in corrinoid production in response to different rates of transient oxygenation is of broad ecological interest because corrinoids are required by many organisms, yet are synthesized de novo only by select microorganisms (e.g., *Pelosinus*) [81]. We postulate that corrinoids produced by *Pelosinus* may also be important for electron mediation between the cell and Fe-(oxyhydr)oxides in soils, considering that pure corrinoids can act as effective electron mediators [82]. Hypothetically, corrinoids in the slow oxygenation treatment could have helped to catalyze the transfer of electrons to more crystalline Fe(III)-(oxyhydr)oxides or shuttled electrons to more available/reactive Fe(III) sites in our study. The high relative expression of *Pelosinu*s genes that share homology with *pgaA* (Fig. 6) indicates that extracellular matrix formation is important during Fe(III)-reducing conditions in soil, which is consistent with recent laboratory culture experiments [68, 69]. This is further supported by our data that suggest genes encoding other extracellular features of *Pelosinus*, such as S-layer complexes and adhesins, are regulated by changes in Fe crystal order (Figs. 3 and 6). Further, the expression of type-IV secretion, surface antigen and bacteriocin genes (Figs. 3 and 6) indicates that specific pathogenic interactions might confer a competitive advantage to *Pelosinus* under Fe(III)-reducing conditions, which could help explain the predominance of *Pelosinus* in many anaerobic and heavy-metal-contaminated environments [66, 83, 84]. Interestingly, the differential expression of the *Pelosinus* bacteriocin gene in our study, a gene that putatively encodes an anti-microbial toxin against other closely related taxa, was significantly higher in the fast oxygenation treatment compared to the slow treatment (Fig. 3), perhaps indicating an increase in direct competition between *Pelosinus* and other Fe(III) reducers during reduction of more abundant SRO Fe(III)-(oxyhydr)oxides following rapid oxygenation.

### The microbiome of anoxic soil is regulated by transient oxygenation events

**Fig. 7:**
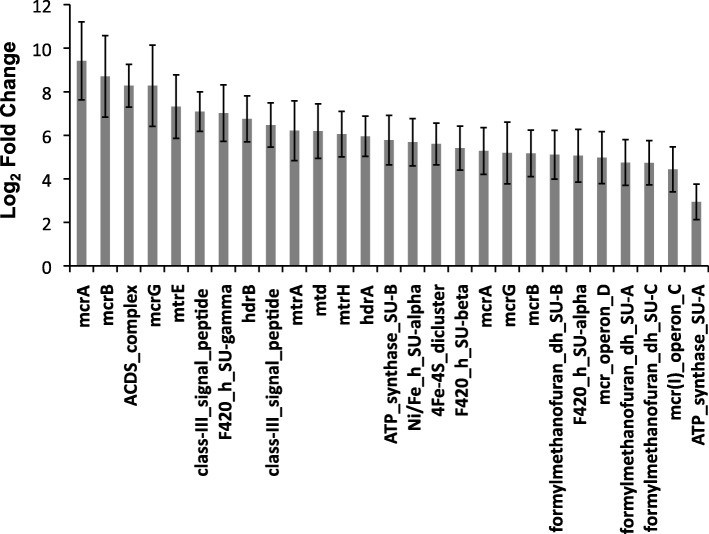
Top differentially expressed genes of *Methanobacterium* detected at significantly higher levels in the fast oxygenation treatment (DESeq; *P* < 0.01). Bars indicate means (± s.e.) (*n* = 3). A complete RefSeq accession-to-annotation key is given in Additional file 3: Table S2

Overall, oxygenation patterns caused distinct metatranscriptomic profiles despite samples being anoxic for 90% of the incubation time and the collection of RNA occurring after a final 7-day period of anoxic conditions (Fig. 2 and Additional file 1: Figures S3–S5). We found that the relative transcript abundance for archaeal CH_4_-producing *Methanobacterium* was significantly higher in the fast oxygenation treatment compared to the slow treatment and was the only primary methanogenic genus that ranked in the top 50 transcript-recruiting genomes (Additional file 1: Figure S3). Species assignments included *M. arcticum*, *M*. *formicicum*, *M*. *lacus*, *M. paludis*, and strain SMA-27. All diagnostic *Methanobacterium* genes required for the H_2_/CO_2_ pathway to CH_4_ (i.e., hydrogenotrophic methanogenesis) were expressed significantly higher in the fast oxygenation treatment (Fig. 7), including those encoding acetyl-CoA dehydrogenase/synthase (ACDS complex) and methyl coenzyme M reductase subunits (*mcrABG*) (Fig. 7). Transcripts for two class (III) signal peptide genes putatively involved in the synthesis of type-IV pilus-like structures were also significantly enriched in the fast oxygenation treatment with similar log_2_ fold changes as the essential H_2_/CO_2_ to CH_4_ pathway genes. This finding suggests that the rate of O_2_ influx during transient oxygenation events regulates both the timing and biological pathway of methanogenesis occurring under predominantly anoxic conditions in soil microbiomes. There was also evidence that different cytochrome oxidases may have been selected to mitigate oxidative stress under different oxygenation conditions, with significantly higher expression of cytochrome *o* ubiquinol oxidase SU2 transcripts in the slow treatment and of cytochrome *c cbb*_*3*_-type oxidase SU1 transcripts in the fast treatment (Additional file 1: Figure S5). Similar enzymes have been shown to be important during redox transitions [31] and during reduction of insoluble metal oxides [65]. We further speculate that production of these enzymes under anoxic conditions in a redox-dynamic system may poise cells to tolerate transient oxygenation events [31, 85, 86].

## Conclusions

We found that the rate of O_2_ delivery during transient oxygenation events in anoxic soil suspensions can influence Fe(III) crystallinity and the expression of specific genes required for Fe(III) reducers to transfer electrons, interact with surfaces, and interact with other cells. Importantly, these data suggest that soil Fe(III) reducers regulate gene expression in response to small differences in Fe-(oxyhydr)oxide crystallinity induced by different oxygenation rates. Overall, our work demonstrates that transient oxygenation events have the potential to direct specific anaerobic pathways and the biogeochemical cycling of Fe and C in humid forest soils. Similar transient oxygenation events are expected to occur in many natural environments, where dynamic changes in water-table depth, irrigation practices, or precipitation can drive redox-dynamic conditions. More broadly, our data suggest that transient oxygenation events in low-O_2_ environments may have global importance due to their effect on key microbial processes that hinge on Fe cycling, including C turnover rates, greenhouse gas emissions, and nutrient availability.

## Additional files

Additional file 1: Supplemental text and figures that give additional information on experimental design and metatranscriptomics (section 1), and Mössbauer spectroscopy (section 2). (PDF 2285 kb)

Additional file 2: **Table S1.** Gives an accounting of sequence reads through processing, reference searching, and binning. (XLSX 45 kb)

Additional file 3: **Table S2.** Lists a key for linking NCBI RefSeq accessions to corresponding functional annotations used in main Figs. 3, 4, 5, 6, and 7. (XLSX 63 kb)

Additional file 4: **Table S3.** Gives Mössbauer parameters for spectra recorded at 140 K. (XLSX 49 kb)

Additional file 5: **Table S4.** Gives Mössbauer parameters for spectra recorded at 77 K. (XLSX 48 kb)

Additional file 6: **Table S5.** Gives Mössbauer parameters for spectra recorded at 4.5 K. (XLSX 48 kb)

Additional file 7: **Table S6.** Gives Mössbauer parameters for spectra recorded at 295 K. (XLSX 47 kb)
