# Supplementary material for: Transient O2 pulses direct Fe crystallinity and Fe(III)-reducer gene expression within a soil microbiome
Source: Microbiome. 2018 Oct 23;6:189. doi: 10.1186/s40168-018-0574-5 (PMC6199725; doi:10.1186/s40168-018-0574-5)
Supplement: Supplementary file 1 — Supplemental text and figures that give additional information on experimental design and metatranscriptomics (section 1), and Mössbauer spectroscopy (section 2). (PDF 2285 kb) [file 40168_2018_574_MOESM1_ESM.pdf]

Supplementary Information

Section 1. Incubation Materials and Methods

1.1 Materials and methods

1.1.1 Sample collection

The Bisley site is part of the NSF funded Long-Term Ecological Research (LTER) and Luquillo Critical Zone Observatory (LCZO) networks. A bulk sample of approximately 1 kg was excavated between 0 and 10 cm depth from an upland valley position [1, 2]. Soil was placed in plastic sampling bags and allowed to air-dry for further processing. Air-dried soil was passed through a 2 mm sieve and homogenized inside of a plastic sample bag before experiments were performed.

1.1.2 Redox-oscillation treatments

During oxygenation, microcosms were removed from the anoxic chamber and 10 ml sterile syringes were fitted with 22 ga, sterile stainless steel needles to inject O_2 (21% in air) into the slow and medium oxygenation microcosms. The appropriate amount of O_2 was added by first injecting to create slight overpressure in each sealed microcosm, pumping the syringe (x3), and removing an equal headspace volume to avoid overpressure after mixing. The fast oxygenation microcosms were exposed to O_2 by hourly removal of caps and stoppers with swirling (x3) in the presence of O_2 (100 ml total exposed and mixed headspace volume), and then resealed with new stoppers and caps. Upon reentry into the anoxic chamber following each 24 hr oxic period, all treatment caps and stoppers were removed and discarded, detectable O_2 levels allowed to clear (fall below detection = 1 ppm in the anoxic chamber), anoxic gas evacuation/fill to restore anoxic conditions, then new stoppers and caps were fitted to begin a new anoxic period.

1.1.3 Incubation sampling scheme

Microcosms under oxic conditions were moved temporarily into the anoxic chamber for sampling, without removing seals, and then all oxic treatments were again transferred outside the chamber to a horizontal shaker. During sampling at all time-points in the anoxic chamber for both oxic and anoxic conditions, to avoid vacuum during sampling and to maintain atm pressure afterward, sealed microcosms were first over-pressured by injection of 1 ml anoxic chamber gas. An equal 1 ml volume of soil suspension was then removed. Micro-centrifuge tubes (2 ml) fitted with rubber o-ring cap seals were used to transfer the suspension aliquots outside the anoxic chamber for centrifugation to separate the aqueous and solid fractions.

1.1.4 Fe(II) extraction and chemical analysis

Each separated aqueous phase was acidified in the anoxic chamber with trace purity 7 M HCl (7 μl/0.5 ml sample) and stored in a clean sealed micro-centrifuge tube in the dark. Trace purity 0.5 M HCl (1 ml) was added to the remaining soil pellet and sealed in the anoxic chamber. The 0.5 M HCl extractions were then vortexed on medium-high speed for 2 hrs in the dark outside the anoxic chamber, after which the extractions were centrifuged as previously described, and the acid supernatant was removed inside the chamber and stored in a clean micro-centrifuge tube in the dark. A modified ferrozine method was used to quantify Fe(II) in acidified aqueous and HCl extracts [3]. Total elemental Fe concentration in the initial soil was determined by lithium(Li)-metaborate fusion and digestion (sub-contracted to Australian Laboratory Services (ALS) Minerals, Reno, NV, USA).

1.1.5 RNA isolation, sequencing and analysis

Soil suspensions collected for RNA analysis at 31 d were transferred inside the anoxic chamber directly from microcosms to Nalgene plastic bottles and sealed with a thick layer of high-vacuum silicone grease applied between the threads of the bottle and cap. The samples were

then immediately frozen in liquid N_2 and subsequently stored at -80°C until further analysis. Samples were prepared for RNA extraction by fracturing each frozen disc of incubated suspension in the bottom of storage bottles that were embedded in dry ice. A ceramic rod was used to fracture the frozen samples, and forceps were used to transfer frozen fragments to bead tubes (Mo Bio, Carlsbad, CA, USA) on dry ice. All equipment, gloves and surfaces were appropriately flamed, wiped with 70% EtOH and sprayed with RNaseZap (Sigma-Aldrich, St. Louis, MO, USA) during all procedures. We chose to use only the slow and fast oxygenation samples for downstream RNA sequencing and analysis as these treatments likely represented the most informative biogeochemical differences (see also main text Methods and Results sections for additional details). We used the medium oxygenation samples for evaluation during preliminary extraction trials. Total RNA was extracted from soils using the RNA PowerSoil® Total RNA Isolation Kit (Mo Bio) following the manufacturer's instructions. In trial runs using the medium oxygenation replicates, different protocols comparing both separated and intact soil suspensions were used to establish the best procedure modifications to extract RNA from the samples containing both aqueous buffer and soil. We found that the most effective extraction strategy, giving the purest high-yield RNA, was to remove the aqueous phase by cold centrifugation and extract the remaining pellet (1 g dry mass equivalent extraction^{-1}) (data not shown). Accordingly, both the fast and slow treatment replicates began the extraction process on dry ice after the frozen fragments had been transferred to bead tubes as described above. A stream of N_2 was used to flush the headspace of each bead tube and then capped to prevent any dramatic redox changes in the steps that followed. Beginning extraction, the sealed N_2-flushed tubes were quickly moved between ice and brief vortexing to initiate thawing. The samples were then immediately placed in a 0°C centrifuge and spun at 4,000 x rcf for 7 min. Removal of the

separated aqueous phase after centrifugation marked the immediate transition to step 1 of total RNA extraction as detailed in the RNA PowerSoil® kit user protocol. All mass transfers that occurred during extraction were recorded during the procedure to calculate RNA yields on a dry soil basis. Initial RNA yields and purity were measured on a NanoDrop spectrophotometer.

The Bacteria Ribo-Zero Magnetic Kit (Epicentre-Illumina, Madison, WI, USA) was then used to remove ribosomal RNA (rRNA), considering rRNA can make up more than 90% of prokaryotic RNA fractions [4-6]. Linear amplification of the re-suspended prokaryotic-rRNA-depleted samples was performed using the MessageAmp II-Bacteria Kit (Ambion® (Thermo Fisher Scientific), Waltham, MA, USA). Fragment size analysis and integrity of the mRNA-amplified samples were checked on a TapeStation 2200 (Agilent Technologies, Santa Clara, CA, USA). Sequence cDNA libraries were constructed, following quality control assessment on a BioAnalyzer 2100 (Agilent), using the KAPA Stranded RNA-Seq kit (Kapa Biosystems, Wilmington, MA, USA) with TruSeq adapters (Illumina, San Diego, CA, USA). Libraries were pooled and sequenced on four lanes using the NextSeq platform (Illumina) to generate 150 nt paired-end sequences. Library construction, process quality control and sequencing were performed at the Georgia Genomics Facility, Athens, GA, USA.

Sequence quality was evaluated using the program FastQC. PEAR was used to merge paired-end sequences [7], and Prinseq was used to trim poly-A/T tails ≥15 nt added in the RNA amplification procedure [4]. RiboPicker was used to remove rRNA sequences (16S, 23S, 18S, 28S, 5S and 5.8S units) using a standalone non-redundant rRNA database (rrnadb) which included the most current versions of SILVA, Ribosomal Database Project RDP-II, GreenGenes, NCBI archeal/bacterial complete genomes rRNA and Rfam databases. Alignments of the remaining sequences were performed with Diamond-sensitive-*BLASTX* searches (bit score ≥40,

E-value $\leq 10^{-3}$) against the NCBI RefSeq protein database [8]. MEGAN6 software was used to bin putative mRNA sequences taxonomically (RefSeq) and functionally (Interpro), and was also used to perform principal coordinates analysis (PCoA) [4, 9]. Distance matrices for PCoA analysis were calculated using Bray-Curtis distances in MEGAN6. Sequences surviving each step of processing are given in Additional file 2: Table S1. Significant differences in relative gene expression between treatments were measured using DESeq2, which included all putative mRNA sequences. Statistical values are those reported by DESeq2 using an adjusted *P* cutoff of 0.1. The primary data handling and analyses were performed on a Linux cluster at the Georgia Advanced Computing Resource Center, Athens, GA, USA.

Section 1. Figures

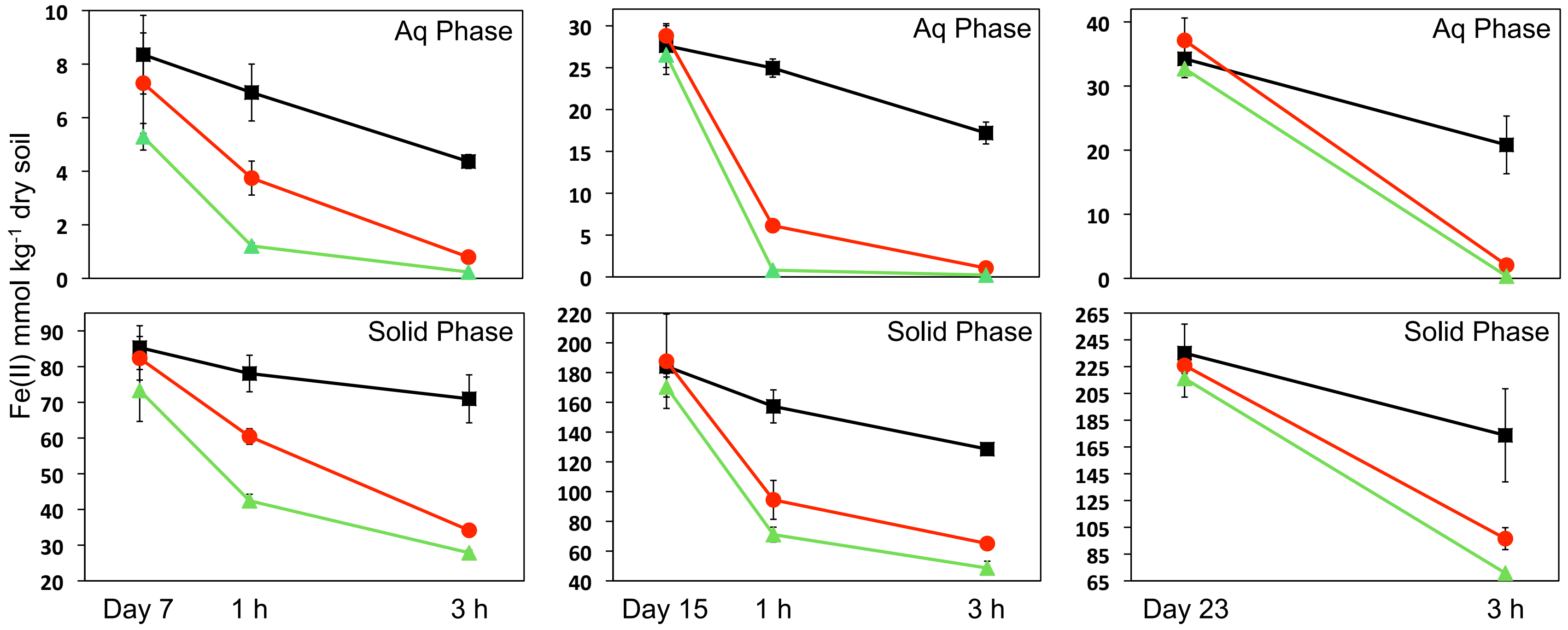

SI Figure S1 Expanded oxic intervals during incubation. Amount of Fe(II) extracted from the aqueous and solid phases when $[O_2]_{atm}$ (21% O_2 in air) was supplied at 2.1 x 10^{-4} (SlowOx; black square), 10^{-3} (MedOx; red circle) or 10^{-2} (FastOx; green triangle) mol hr^{-1}. Data points are means (±s.d.) (n=3). Each plot column corresponds to a 3 h oxic interval beginning at 7 d, 15 d or 23 d that are denoted by light grey vertical bars in main Figure 1a.

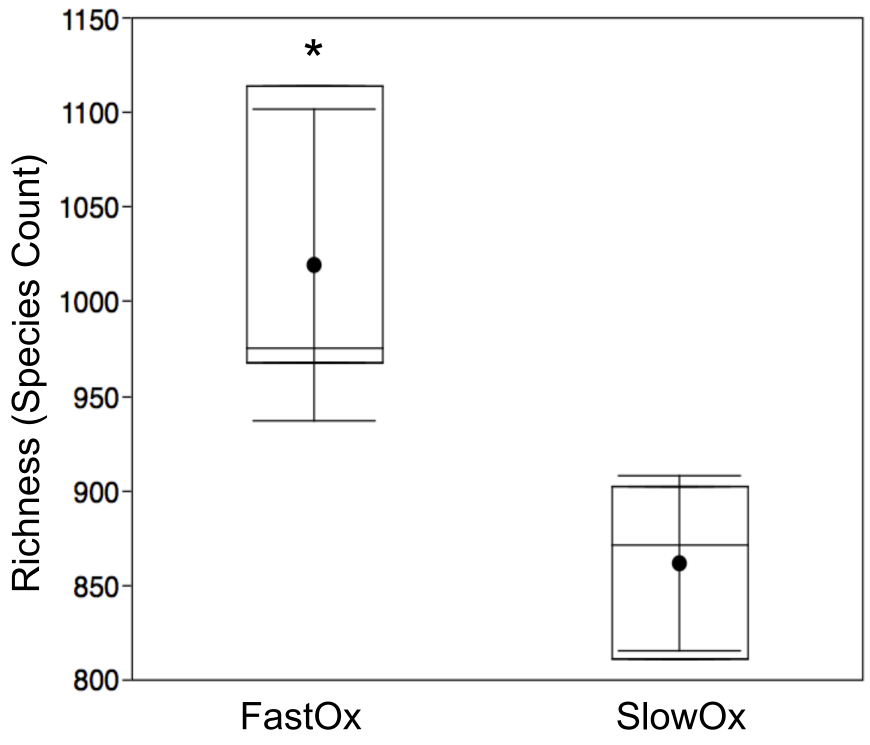

SI Figure S2. Species richness for the fast and slow oxygenation treatments based on binning of transcripts to reference genomes. Central dots and error bars represent means (±s.d.) (n=3). Top and bottom edges of the boxes and horizontal line within boxes show individual replicate values. T-test significance: *=*P*<0.05.

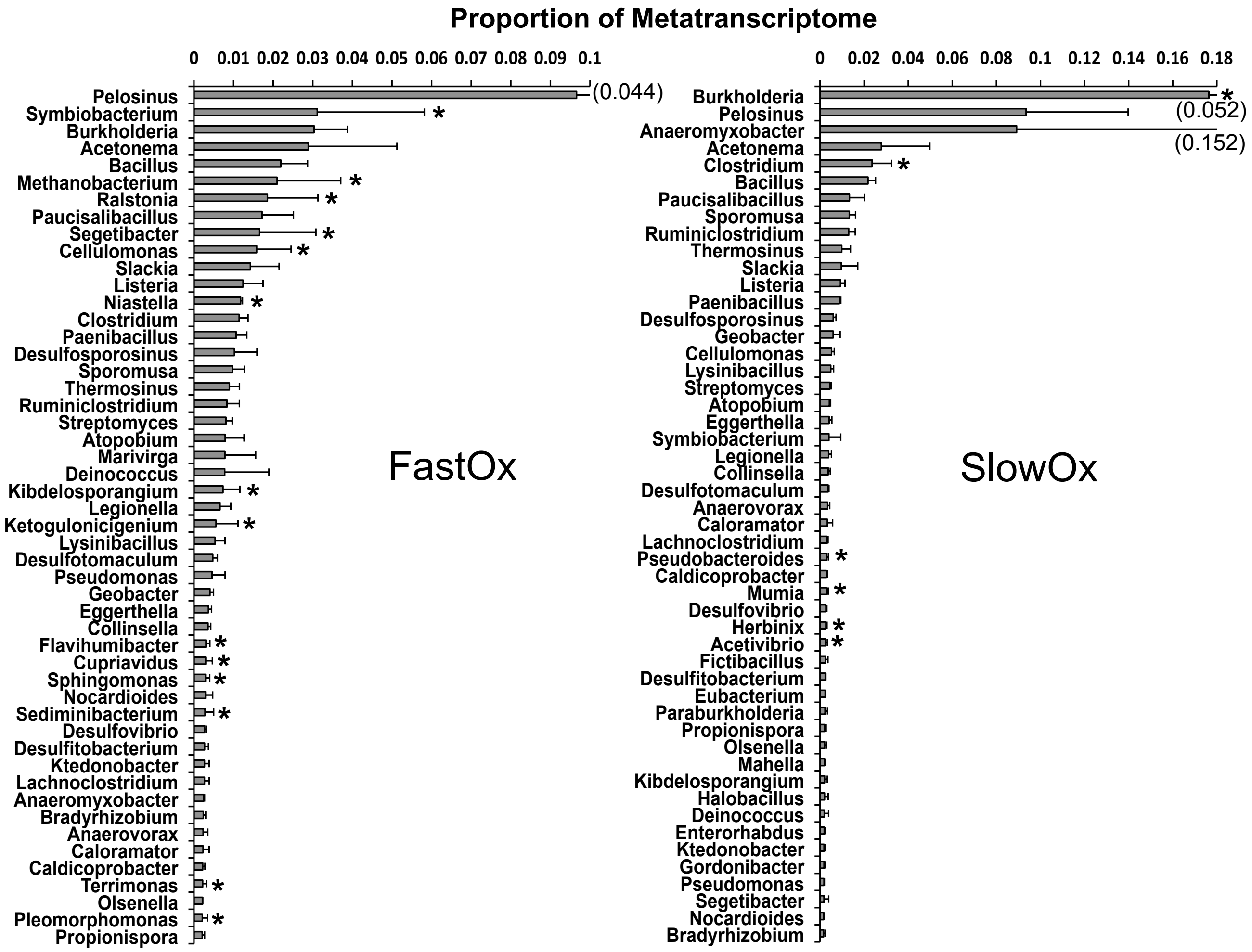

SI Figure S3. Top 50 taxa in terms of relative contribution to the metatranscriptome. Bars indicate means (±s.e.) (n=3). Where error bars extend beyond the graphed range, the standard deviation is given in parentheses. Significant differences are indicated by asterisks (DESeq; $P<0.05$).

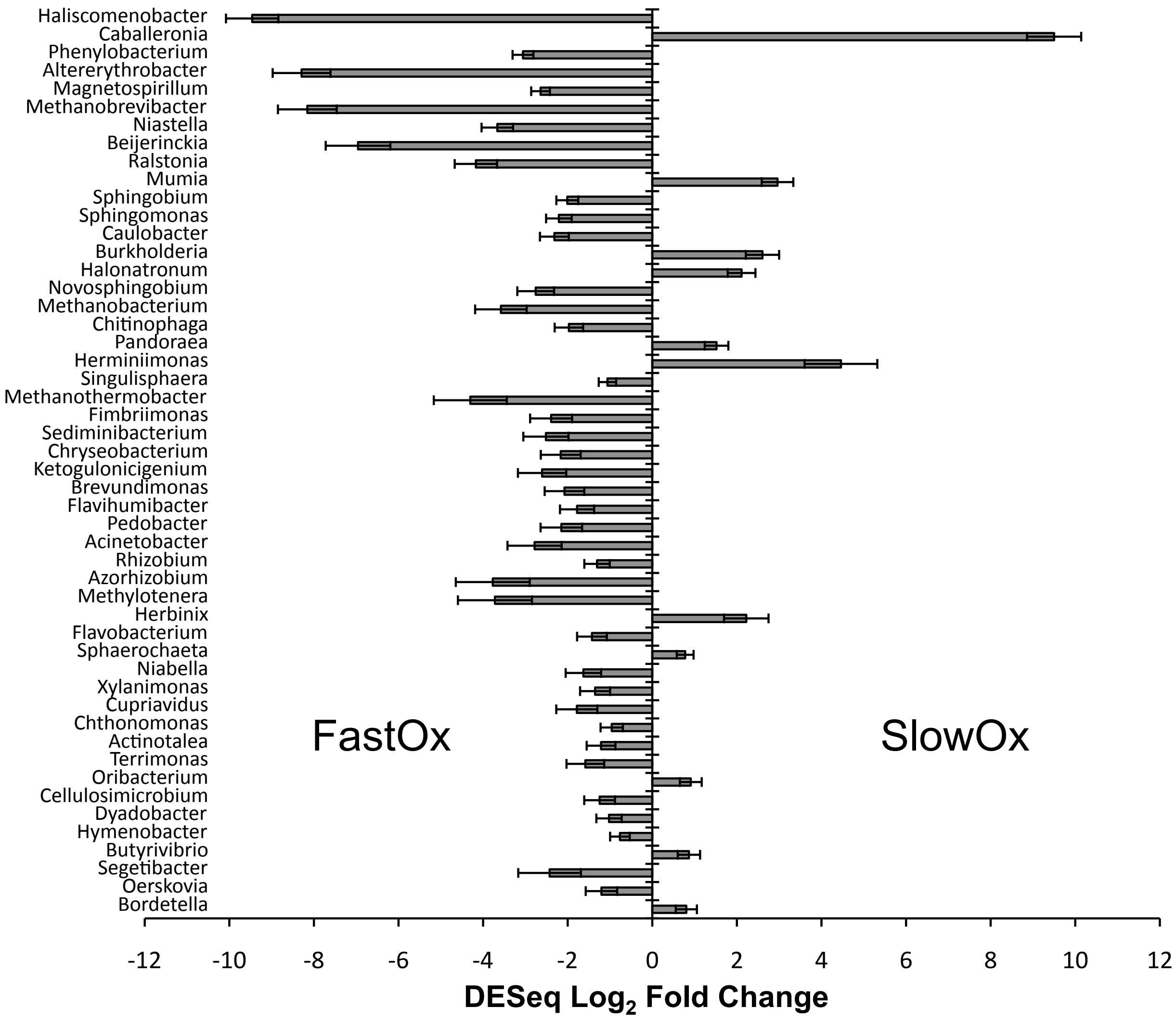

SI Figure S4 Top 50 significantly different $\log_2$ fold changes for transcripts assigned to genera in the fast and slow oxygenation treatments. Data are shown as means (±s.e.) (n=3).

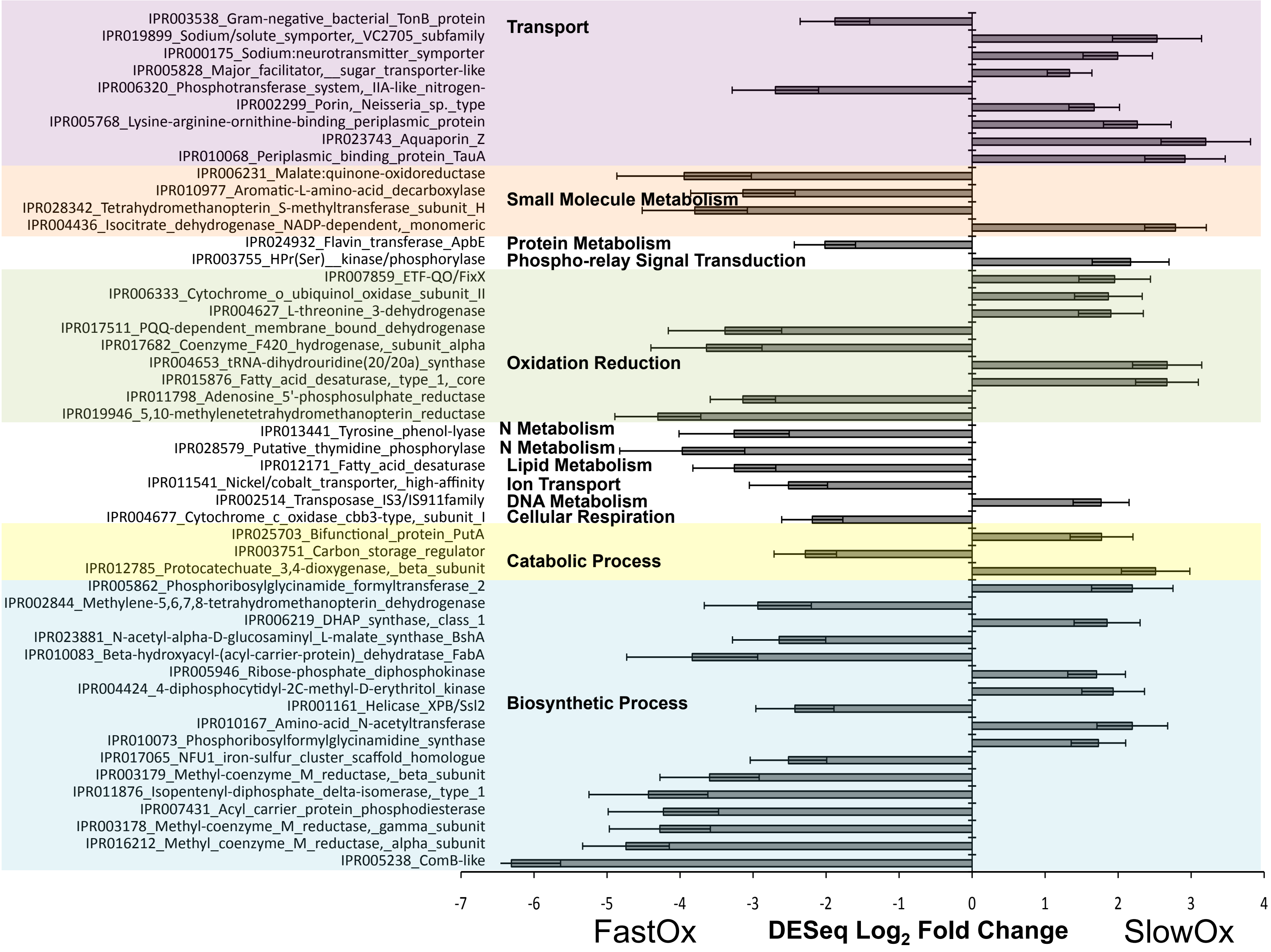

IPR003538_Gram-negative_bacterial_TonB_protein
IPR019899_Sodium/solute_symporter,_VC2705_subfamily
IPR000175_Sodium:neurotransmitter_symporter
IPR005828_Major_facilitator,__sugar_transporter-like
IPR006320_Phosphotransferase_system,_IIA-like_nitrogen-
IPR002299_Porin,_Neisseria_sp._type
IPR005768_Lysine-arginine-ornithine-binding_periplasmic_protein
IPR023743_Aquaporin_Z
IPR010068_Periplasmic_binding_protein_TauA
IPR006231_Malate:quinone-oxidoreductase
IPR010977_Aromatic-L-amino-acid_decarboxylase
IPR028342_Tetrahydromethanopterin_S-methyltransferase_subunit_H
IPR004436_Isocitrate_dehydrogenase_NADP-dependent,_monomeric
IPR024932_Flavin_transferase_ApbE
IPR003755_HPr(Ser)__kinase/phosphorylase
IPR007859_ETF-QO/FixX
IPR006333_Cytochrome_o_ubiquinol_oxidase_subunit_II
IPR004627_L-threonine_3-dehydrogenase
IPR017511_PQQ-dependent_membrane_bound_dehydrogenase
IPR017682_Coenzyme_F420_hydrogenase,_subunit_alpha
IPR004653_tRNA-dihydrouridine(20/20a)_synthase
IPR015876_Fatty_acid_desaturase,_type_1,_core
IPR011798_Adenosine_5'-phosphosulphate_reductase
IPR019946_5,10-methylenetetrahydromethanopterin_reductase
IPR013441_Tyrosine_phenol-lyase
IPR028579_Putative_thymidine_phosphorylase
IPR012171_Fatty_acid_desaturase
IPR011541_Nickel/cobalt_transporter,_high-affinity
IPR002514_Transposase_IS3/IS911family
IPR004677_Cytochrome_c_oxidase_cbb3-type,_subunit_I
IPR025703_Bifunctional_protein_PutA
IPR003751_Carbon_storage_regulator
IPR012785_Protocatechuate_3,4-dioxygenase,_beta_subunit
IPR005862_Phosphoribosylglycinamide_formyltransferase_2
IPR002844_Methylene-5,6,7,8-tetrahydromethanopterin_dehydrogenase
IPR006219_DHAP_synthase,_class_1
IPR023881_N-acetyl-alpha-D-glucosaminyl_L-malate_synthase_BshA
IPR010083_Beta-hydroxyacyl-(acyl-carrier-protein)_dehydratase_FabA
IPR005946_Ribose-phosphate_diphosphokinase
IPR004424_4-diphosphocytidyl-2C-methyl-D-erythritol_kinase
IPR001161_Helicase_XPB/Ssl2
IPR010167_Amino-acid_N-acetyltransferase
IPR010073_Phosphoribosylformylglycinamidine_synthase
IPR017065_NFU1_iron-sulfur_cluster_scaffold_homologue
IPR003179_Methyl-coenzyme_M_reductase,_beta_subunit
IPR011876_Isopentenyl-diphosphate_delta-isomerase,_type_1
IPR007431_Acyl_carrier_protein_phosphodiesterase
IPR003178_Methyl-coenzyme_M_reductase,_gamma_subunit
IPR016212_Methyl_coenzyme_M_reductase,_alpha_subunit
IPR005238_ComB-like
Transport
Small Molecule Metabolism
Protein Metabolism
Phospho-relay Signal Transduction
Oxidation Reduction
N Metabolism
N Metabolism
Lipid Metabolism
Ion Transport
DNA Metabolism
Cellular Respiration
Catabolic Process
Biosynthetic Process
-7 -6 -5 -4 -3 -2 -1 0 1 2 3 4
FastOx
DESeq Log2 Fold Change
SlowOx

SI Figure S5 Top 50 significantly different $\log_2$ fold changes based on transcript assignments to Interpro families and grouped by Gene Ontology terms in distinctly shaded areas for the fast and slow oxygenation treatments. Data are shown as means (±s.e.) (n=3).

Section 2. ^{57}Fe Mössbauer Spectroscopy

2.1 Introduction

We employ ^{57}Fe Mössbauer spectroscopy to compare Fe site populations and to assess changes in Fe mineral crystallinity between treatments. The initial soil used in this study is similar in origin to the soil used in Ginn *et al.* [10], Tishchenko *et al*. (2015), and Ginn *et al.* [11]. A full description of the mineral composition of that soil is contained in the electronic annex of Tishchenko et al (2015). The soils used here were excavated from a different landscape position within the Bisley Watershed [1, 2].

2.2 Methods

2.2.1 Mössbauer collection and analysis methods

Samples collected under oxic conditions for Mössbauer spectroscopy at 24 d during the incubation were centrifuged as previously described for sample aliquots, and the supernatants subsequently removed in the anoxic chamber. Soil pellets remaining in o-ring-lid micro-centrifuge tubes were sealed under anoxic headspace and stored at -80°C until further analysis. For analysis, frozen samples were transferred to the anoxic chamber to be prepared as sealed mounts for Mössbauer spectroscopy measurements. For each treatment, replicate soil gels (n=3) were mixed (i.e., pooled) (180 mg total equivalent dry mass mount^{-1}) within the cavity of a thin nylon ring and sealed between two layers of Kapton tape. Mössbauer spectroscopy measurements always began immediately at 4.5 Kelvin (K) to mitigate potential changes in mineralogy.

Absorption spectra of the initial air-dried soil and sampled incubation soils were collected in transmission mode with a variable temperature He-cooled cryostat (Janis Research Co.) and a 1024 channel detector. A ^{57}Co source (~50 mCi) embedded in a Rh matrix was used at room

temperature. Velocity (i.e., gamma-ray energy) was calibrated using α-Fe foil at 295K and all center shift (CS) and peak positions are reported with respect to this standard. The transducer was operated in constant acceleration mode and spectra were folded to 512 channels to achieve a flat background. Mössbauer spectral fitting was performed using Recoil^{TM} software (ISA Inc.) with the Voigt-based fitting (VBF) method of Rancourt and Ping [12] for quadrupole splitting distributions (QSDs) and combined hyperfine field distributions (HFDs). The Lorentzian linewidth (HWHM) parameter was held at 0.1425 mm s^{-1}, corresponding to the measured linewidth using α-Fe foil at 295K on our instrument. All Mössbauer parameter definitions and a description of the relevant notation are given in Rancourt and Ping [12].

2.2.2 Mössbauer spectral analysis approach

Fitting of all Mössbauer spectra was performed using the Voigt-based fitting (VBF) method of Rancourt and Ping [13] for quadrupole splitting distributions (QSDs) and combined hyperfine field distributions (HFDs), as implemented in the Recoil^{TM} software, ISA Inc. (http://www.isapps.ca/ recoil). All VBF Mössbauer parameter definitions and a description of the relevant notation are given by Rancourt and Ping [13]. All errors in Mössbauer fitting parameters are two-standard deviation (2σ) errors, as calculated by Recoil^{TM}. In reporting quantitative phase abundances or site populations, it is assumed that the Mössbauer recoilless fractions of all detected phases or Fe-bearing components are equal, such that subspectral areas (expressed as fractions of total spectral area) are equal to the amounts of Fe (expressed as fractions of total Fe) in the corresponding phases or components. This assumption is expected to be valid at cryogenic temperatures [14, 15]. We left most fitting parameters, those typically used to fine-tune the fit, at their general, conservative values during modelling in Recoil. We disallowed parameter coupling between the center shift (CS), quadrapole splitting (QS for doublets or ε for sextets) and average

hyperfine field (Bhf) and constrained the area ratios between the doublet lines to 1:1 and area ratios between sextet lines 2 and 3 to 2:1 and lines 1 and 3 to 3:1.

2.2.3 Mössbauer spectral fitting approach

In Mössbauer spectroscopy, each spectral signal (or set of related signals) corresponds to one Fe-bearing solid phase or to a group of unresolved Fe-bearing solid phases. These signal features take the form of a doublet, sextet, octet (none resolved here) or a collapsed sextet—indicating a solid-phase near its magnetic ordering temperature (T_N or T_C). Solid-phases well above (doublet) or below (sextet) their T_N will not exhibit any vertical (i.e., count axis) distance between the peak troughs and the baseline. When Fe solid-phases are near their T_N, they exhibit an intermediate shape between a doublet and full sextet, which fills the area between the upper baseline and the inverse troughs of the peaks. We approximate this by using a separate collapsed sextet feature (i.e., a sextet with exceedingly large line widths and Bhf = 0 T). Assuming equal Mössbauer recoilless fractions (i.e., that the *measurable* gamma rays emitted by each phase are proportional to the phase abundance), the total spectral area of a given signal feature is proportional to the amount of Fe in the corresponding solid phase(s). Furthermore, it can then be assumed that each phase occupies the same total spectral area at cyro-temperatures, although not necessarily the same spectral shape or site. For instance, nano-scale Fe(III)-(oxyhydr)oxide minerals form a doublet at 295K and magnetically order to form a sextet only when cooled sufficiently to prevent thermal disordering of the macroscopic magnetic ordering within the material (Rancourt, 2001).

We used two fitting approaches to help guide our interpretations in this study. The main approach used for all calculations and presented data plots included a slightly modified parameter set (described in SI section 2.2.4 below) compared to that described by Ginn *et al.*

[11] for LCZO soils. The parameters based on Ginn *et al*. (2017) include the Ti-Fe mineral ilmenite that had been implicated previously in LCZO soils (Tishchenko et al., 2015). The Ginn *et al*. (2017) fitting approach was applied in the present study at 77K, 140K and 295K for comparison and reference, and both the main and comparative fitting routines have been denoted in parameter tables (Additional files 4-7: Tables S3-6).

2.2.4 Mössbauer spectral components

Spectral signals include the following in both the main and comparative fitting approaches: (1) an Fe(III) quadrupole doublet (labelled Q-Fe(III)) that can represent Fe(III) in silicates, surface-complexed Fe(III), and/or superparamagnetic Fe(III)-(oxyhydr)oxides; (2) an Fe(II) quadrupole doublet (labelled Q-Fe(II)) that can represent biogenic Fe(II), Fe(II) in silicates, surface-complexed Fe(II), or superparamagnetic Fe(II) solid phases; (3) an Fe(III) sextet (labelled HFD-OxHy) that corresponds to magnetically-ordered Fe(III)-(oxyhydr)oxides; (4) an Fe(II) hyperfine site (labelled HFD-Fe(II)) that displays relatively high CS and ε values compared to a low Bhf, which likely represents Fe(II) at the surface of magnetically ordered (oxyhydr)oxides that have been reduced during microbial Fe(III) reduction, or Fe(II) in poorly crystalline solid phases such as nano-magnetite [16]; (5) a collapsed Fe(III) sextet (labelled HFD-(b)OxHy) that represents Fe(III) in relatively more disordered (oxyhydr)oxides near their blocking temperature [17, 18]. We also use an asymmetric Fe(III) *doublet* (labelled HFD-Fe(III)) in the main fitting routine (Additional files 4-6: Tables S3-5; and SI Figures S6-12). This feature approximates a transition between a quadrupole ferric doublet and a collapsed sextet (HFD-(b)OxHy) [19, 20], and has a large quadrupole-hyperfine perturbation (i.e., ε = -0.6 to -1) relative to a Bhf near 0 T.

2.3. Mössbauer phase analysis of initial and treated soils

Below, we provide an analysis of the Fe populations identified in the samples as recorded at different temperatures. The primary aim of our Mössbauer analysis was to determine changes in crystal order between the treated samples dependent on different oxygenation rates imposed during the experiment.

2.3.1 Fe(III)-(oxyhydr)oxide populations: Changes in Fe crystallinity

Comparison of 140K, 77K, and 4.5K Mössbauer spectra allows us to characterize differences in the crystallinity-continuum of Fe(III)-(oxyhydr)oxides that exist between oxygenation treatments. As collection temperature is lowered, portions of the Fe(III)-(oxyhydr)oxide populations magnetically order and give rise to a sextet (SI Figures S6-8), with the more crystalline portions of the population ordering at higher temperatures. The spectral parameters of each sextet among the samples (*ca* QS = -0.11; Bhf peak = 49 T @ 4.5K) are similar to those reported for nano-goethite standards at 4.2K. Those Fe(III)-(oxyhydr)oxides that are blocked or near their blocking temperature at 4.5K comprise *ca* 21% of the spectral area. We have fit this type of Fe population using a collapsed sextet (HFD-(b)OxHy).

At higher temperatures, such as 140K and 77K, we expect the more-crystalline Fe(III)-(oxyhydr)oxides to display a sextet, while more disordered phases remain as a doublet. We find at 140K that both treated samples have slightly larger sextet areas than the initial air-dried soil (SI Figure S9; Additional file 4: Table S3). This suggests that both treatments contained larger abundances of more crystalline Fe(III)-(oxyhydr)oxides than the initial soil following incubation. We find little difference in total sextet areas at 140K between the treatments. As temperatures decrease, both the treatments and the initial soil display similar total sextet areas as magnetic ordering increases.

Each sextet, regardless of collection temperature, is best fit by using a minimum of two components (i.e., weighted Gaussian components). These individual components represent averaged end-member domains of a continuum of magnetically ordered Fe(III)-(oxyhydr)oxides, with one component approximating Fe that is relatively more crystalline compared to the other component. Comparison of the subpopulation (component) spectral areas within each sextet, at each temperature, can thus be used to characterize the degree of crystal order in each sample. This characterization can further be carried out among treatments analyzed at the same temperature, by calculating and comparing the spectral areas of a common (similar Bhf probability maxima) sextet component between treatments.

By comparing area normalized plots of the total fits for spectra at a given temperature, we find that sextet peaks are relatively more pronounced for the slow oxygenation treatment based on visual inspection alone (SI Figures S9-11). The Bhf probability maxima for 77K sextets (that occur between 45 T and 50 T) increase according to treatment in the order of initial soil < fast oxygenation < slow oxygenation (SI Figure S12). We attribute the more prominent 45-50 T Bhf distribution of the slow oxygenation treatment to a larger subpopulation of *more-crystalline* Fe(III)-(oxyhydr)oxides that magnetically order following exposure to multiple redox cycles. For the initial soil, and the fast and slow oxygenation soils collected 24 d following incubation, 25±1.2, 30.6±1.5 and 38.1±1.1% Fe_{Total} was measured in the more-crystalline pool of FeOOH, respectively (Figure 1b in main text), by calculating and comparing (among samples) prominent 77K sextet component areas that were fit with a single hyperfine site and had a maximum Bhf of *ca* 48 T (i.e., Bhf between 45 and 50 T corresponding to the common probability maximum among samples (SI Figure S12)). Taken together, this supports our hypothesis that slower oxygenation rates lead to increased Fe(III) crystal order during redox cycling.

2.3.2 Paramagnetic and weak-magnetically ordered Fe(III)

Typically, Fe(III) atoms in silicates and organic complexes are too distant from each other to magnetically order. Adsorbed Fe(III) atoms on surfaces may only show magnetic ordering if in close proximity to neighbouring magnetically ordered phases. Substituted Fe(III) in kaolinite [21] typically yields a QS value of 0.52 mm s^{-1}, however, the presence of substituted Fe(II) would likely increase structural distortion and could lead to higher Fe(III) quadropole spliting values [22]. Some Fe(III) in our samples may be associated with the 2:1:1 mineral chlorite as XRD analysis suggests this mineral is present in the clay size fraction [1]. Fe(III) in octahedral positions of Fe-poor 2:1 minerals, or those with significant Fe(II) substitution, also yield larger Fe(III) quadropole splitting values [23]. Overall, however, completely resolving silicate Fe(III) from nano-particulate, superparamagnetic, Fe(III)-(oxyhydr)oxides is not possible in our study. We can only speculate on the possible Fe(III)-bearing phases that are actually represented by the Q-Fe(III) doublet remaining at 4.5K in our samples.

2.3.3 Paramagnetic and weak-magnetically ordered Fe(II)

The high-velocity ferrous doublet line (H_L) at *ca* 2.7 mm s^{-1} is consistent with those commonly reported for aluminosilicate clays [22], including chlorite, which was identified previously in these soils via XRD. The H_L for ferrous substitution in kaolinite averages 2.4 ± 0.1 mm s^{-1} based on a compilation of eight studies reporting Fe(II) in kaolinites [22] and may contribute to our observed peak position. However, adsorbed Fe(II) may also contribute to this peak, as Rancourt et al. [24] have shown that Fe(II) sorbed to bacterial cells can yield H_L lines even above 2.8 mm s^{-1}.

The Fe(II) populations detected in all samples at 4.5K are unique in the fact that we can model the fraction of Fe(II) (HFD-Fe(II)) that displays weak magnetic order, in addition to the

remaining ferrous doublet [16] (SI Figures S6-8). From 77K to 4.5K, each sample shows at least a 50% decrease in Q-Fe(II) spectral area, which is consistent with magnetic ordering of Fe(II). Likely phase identities of the weak-magnetically ordered Fe(II) include nano-magnetite and/or Fe(II) sorbed to magnetically ordered solid phases such as Fe(III)-(oxyhydr)oxides at 4.5K [16]. There are also notable comparisons between the amounts of total Fe(II) quantified by MBS and those measured in 0.5 M HCl extractions. The initial soil contained 72 mmol kg^{-1} total Fe(II) based on MBS analysis, but less than 5 mmol kg^{-1} Fe(II) was extracted by HCl. Following incubation and treatments, the fast oxygenation sample contained 81 mmol kg^{-1} total Fe(II) based on MBS, yet 50 mmol kg^{-1} was extracted by HCl. The amount of total Fe(II) detected by MBS for the slow oxygenation treatment, however, was equal to the corresponding HCl extractable concentration (*ca* 150 mmol kg^{-1} Fe(II)) (Figure 1a in the main text, at 24 d). One explanation for these correlations between extractable and total Fe(II) might be that increased Fe(II)/Fe(III) stoichiometry in the solid phase, following microbial Fe(III) reduction, increases Fe mineral reactivity towards dissolution in 0.5 M HCl.

2.3.4 Comparative Fits

We also performed comparative Mössbauer fitting of the 77K and 140K spectra, including a fit of the initial soil spectrum collected at 295K for reference, using similar modelling parameters as those used in Ginn *et al*. (2017) for analyzing LCZO soils. These comparative fits are largely in agreement with those described above in terms of the components, while the comparative fitting does not include the transition feature HFD-Fe(III) that was included in the 77K and 140K spectra for the main fits. The comparative fits include the mineral ilmenite and the collapsed sextet site, which has been used in prior fitting routines published on similar soils (Tishchenko *et al*., 2015; Ginn *et al*., 2017).

The comparative fits also indicate increases in the degree of crystallinity between samples (Additional files 4-5: Table S3-4). While the main fits find the sharpest component of the sextets increased in spectral area according to treatment, the comparative fits—which have similar areas for the sharpest sextet components across samples—find that the average sextet width increases from the initial soil (44T) to the fast oxygenation (45.9T) to the slow oxygenation (46.7T) treatments. Increasing sextet widths, corresponding to greater hyperfine field strength (Bhf), is strongly correlated with increasing crystallinity in Fe(III)-(oxyhydr)oxide phases [25].

Section 2. Figures

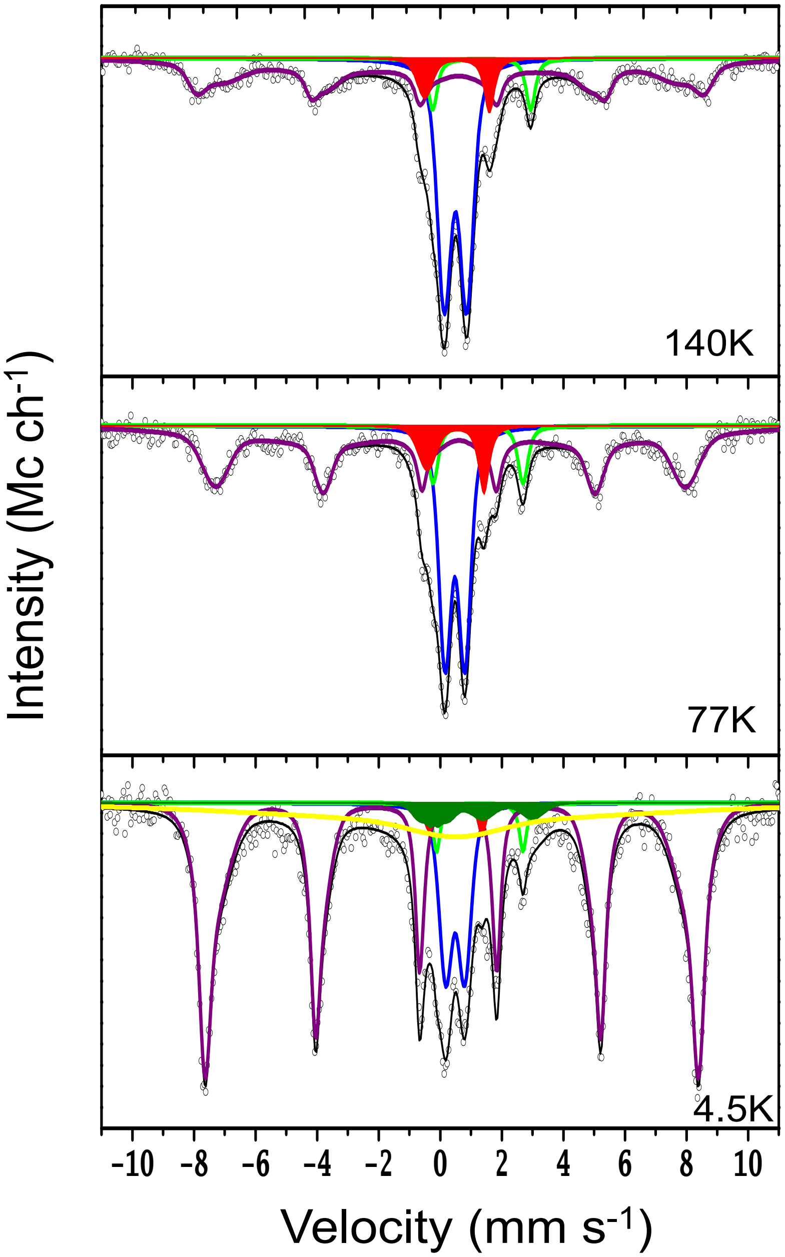

SI Figure S6 ^{57}Fe Mössbauer spectra of the air-dried **initial soil** at 140K, 77K, and 4.5K before incubation. In each spectrum, the black solid line is the total calculated fit, through the discrete data points (open circles). The resolved spectral components and assignments are: (1) Q-Fe(III), the deep central doublet corresponding to paramagnetic/superparamagnetic Fe(III) (blue line); (2) Q-Fe(II), the ferrous doublet corresponding to paramagnetic/superparamagnetic Fe(II) (green line); (3) HFD-Fe(III), the asymmetric *doublet* corresponding to Fe(III) in transition between a ferric quadrupole doublet and collapsed sextet (red fill); (4) HFD-OxHy, the dominant sextet corresponding to Fe(III)-(oxyhydr)oxides that are magnetically ordered (purple line); (5) HFD-(b)OxHy, the collapsed 'sextet' (yellow line); and (6) HFD-Fe(II), Fe(II) that displays weak magnetic ordering at 4.5K (dark green fill).

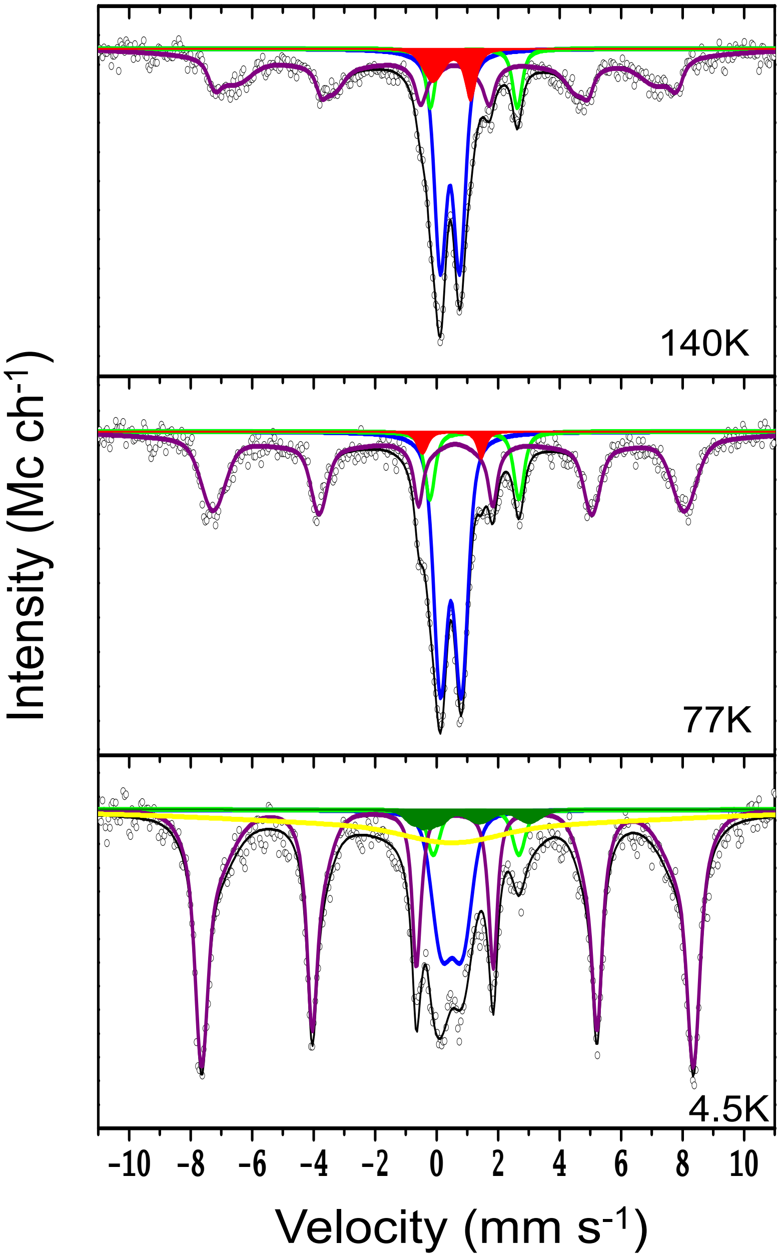

SI Figure S7 ^{57}Fe Mössbauer spectra of the incubated **fast oxygenation** treated soil at 140K, 77K, and 4.5K following incubation. In each spectrum, the black solid line is the total calculated fit, through the discrete data points (open circles). The resolved spectral components and assignments are: (1) Q-Fe(III), the deep central doublet corresponding to paramagnetic/superparamagnetic Fe(III) (blue line); (2) Q-Fe(II), the ferrous doublet corresponding to paramagnetic/superparamagnetic Fe(II) (green line); (3) HFD-Fe(III), the asymmetric *doublet* corresponding to Fe(III) in transition between a ferric quadrupole doublet and collapsed sextet (red fill); (4) HFD-OxHy, the dominant sextet corresponding to Fe(III)-(oxyhydr)oxides that are magnetically ordered (purple line); (5) HFD-(b)OxHy, the collapsed 'sextet' (yellow line); and (6) HFD-Fe(II), Fe(II) that displays weak magnetic ordering at 4.5K (dark green fill).

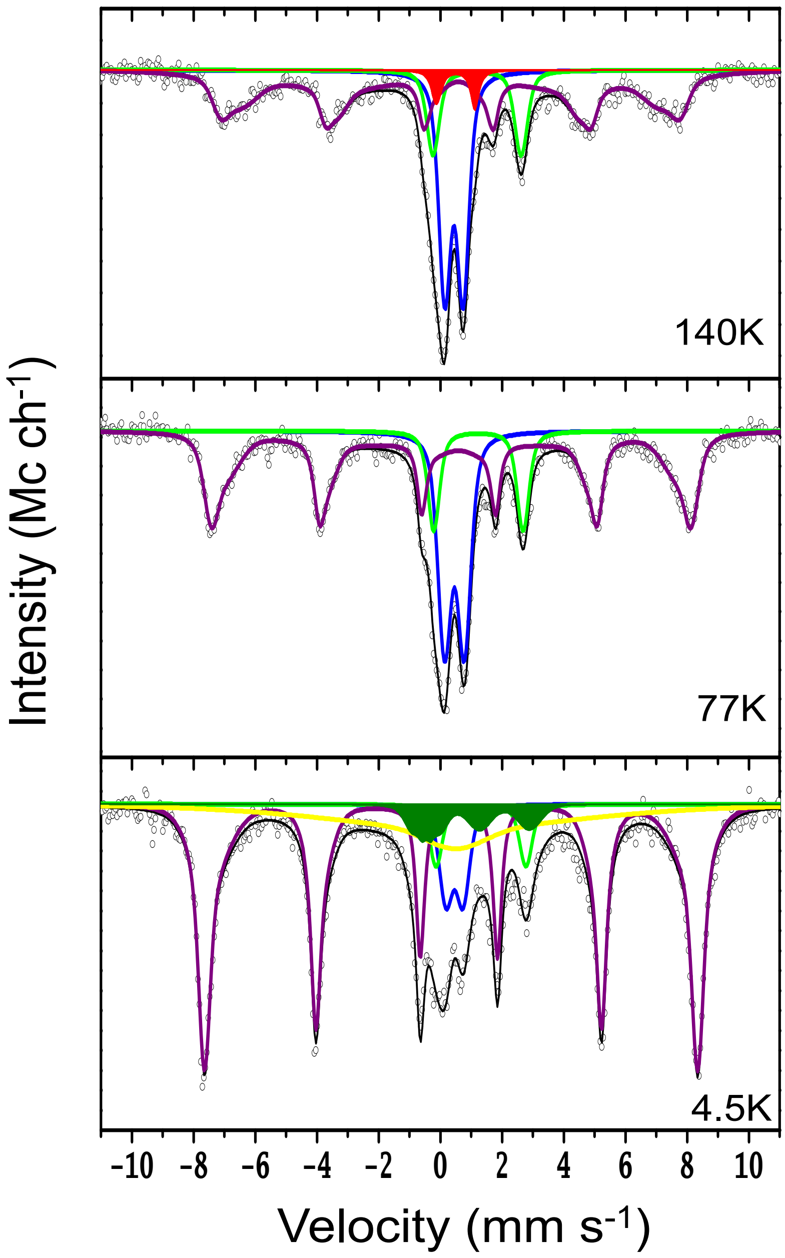

SI Figure S8 ^{57}Fe Mössbauer spectra of the incubated **slow oxygenation** treated soil 140K, 77K, and 4.5K before incubation. In each spectrum, the black solid line is the total calculated fit, through the discrete data points (open circles). The resolved spectral components and assignments are: (1) Q-Fe(III), the deep central doublet corresponding to paramagnetic/superparamagnetic Fe(III) (blue line); (2) Q-Fe(II), the ferrous doublet corresponding to paramagnetic/superparamagnetic Fe(II) (green line); (3) HFD-Fe(III), the asymmetric *doublet* corresponding to Fe(III) in transition between a ferric quadrupole doublet and collapsed sextet (red fill); (4) HFD-OxHy, the dominant sextet corresponding to Fe(III)-(oxyhydr)oxides that are magnetically ordered (purple line); (5) HFD-(b)OxHy, the collapsed 'sextet' (yellow line); and (6) HFD-Fe(II), Fe(II) that displays weak magnetic ordering at 4.5K (dark green fill).

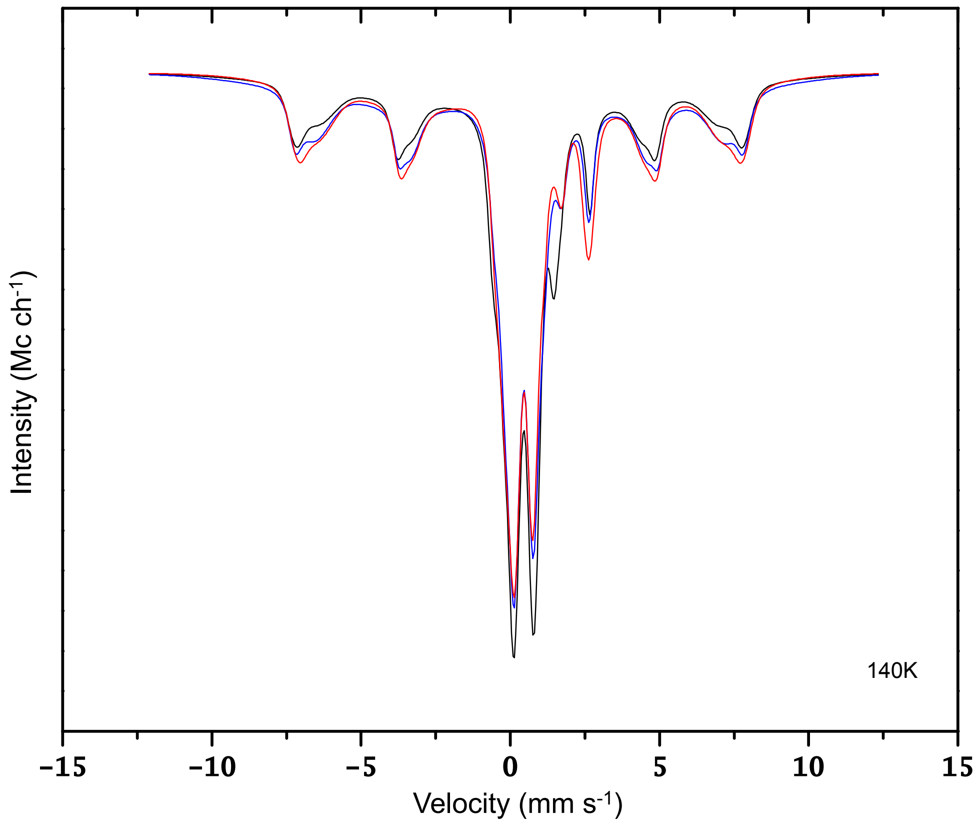

SI Figure S9 Area normalized ^{57}Fe Mössbauer total fits of the air-dried **initial soil** (black line), **fast oxygenation treated soil** (blue line) and **slow oxygenation treated soil** (red line) at 140K. Relative comparisons show that both treated samples have more prominent sextets compared to that of the initial soil, and the slow oxygenation treatment has the most prominent sextet of all. This suggests a larger abundance of more-crystalline Fe(III)-(oxyhydr)oxides that magnetically order in the slow oxygenation treated soil.

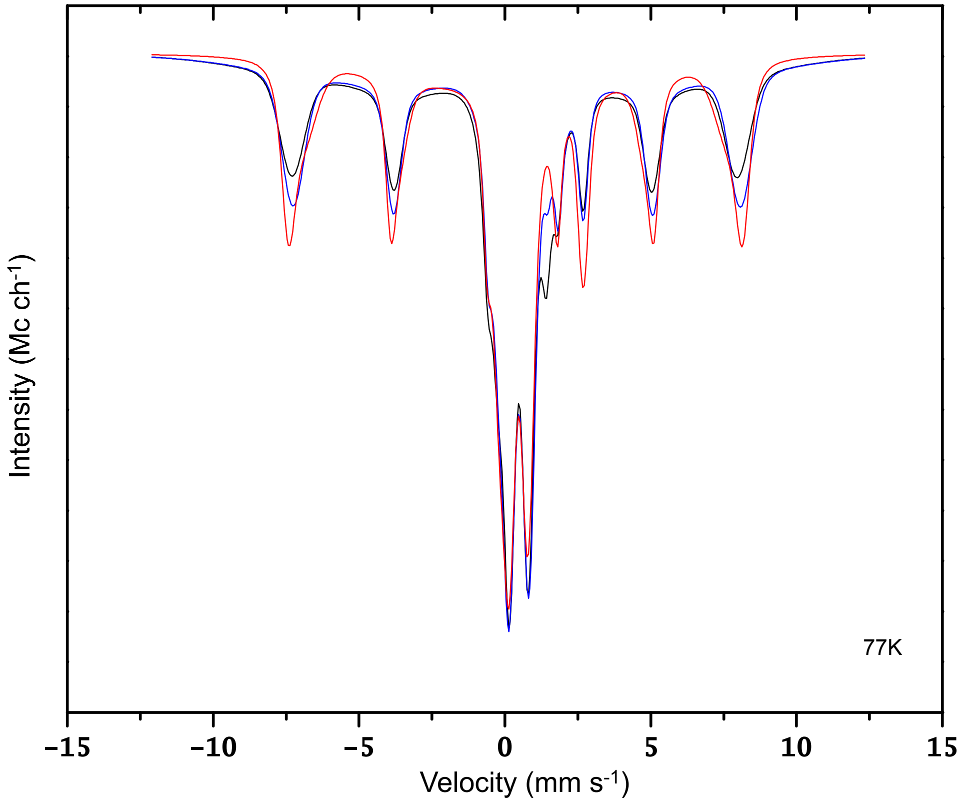

SI Figure S10 Area normalized ^{57}Fe Mössbauer total fits of the air-dried **initial soil** (black line), **fast oxygenation treated soil** (blue line) and **slow oxygenation treated soil** (red line) at 77K. Relative comparisons show that both treated samples have more prominent sextets compared to that of the initial soil, and the slow oxygenation treatment has the most prominent sextet of all. This suggests a larger abundance of more-crystalline Fe(III)-(oxyhydr)oxides that magnetically order in the slow oxygenation treated soil.

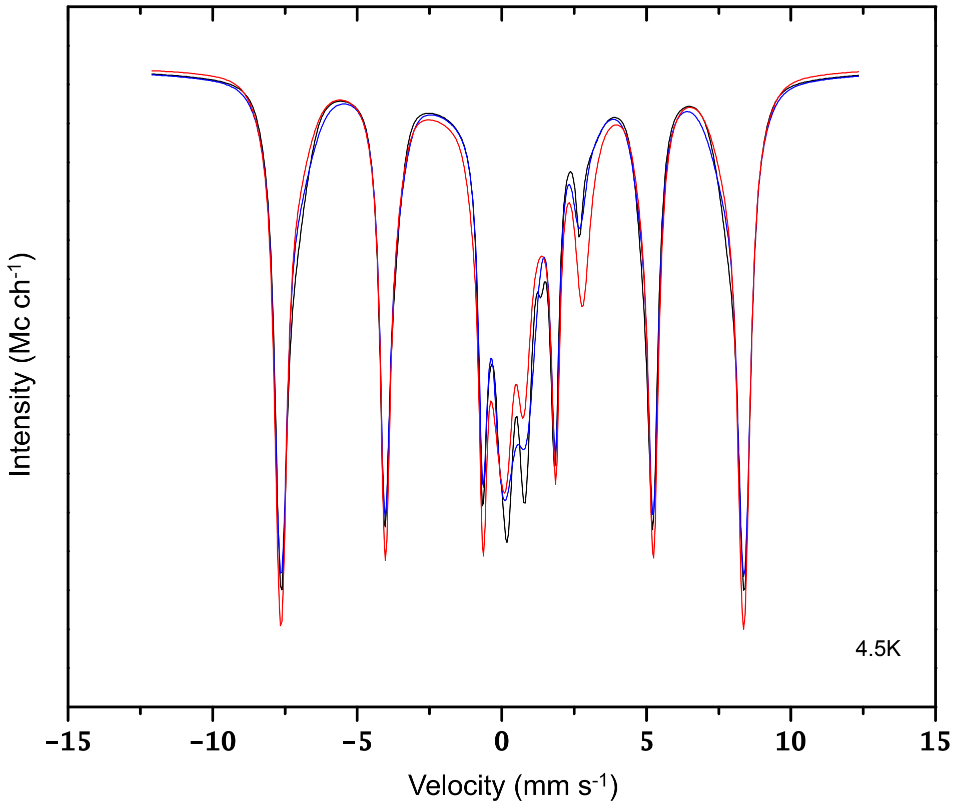

SI Figure S11 Area normalized ^{57}Fe Mössbauer total fits of the air-dried **initial soil** (black line), **fast oxygenation treated soil** (blue line) and **slow oxygenation treated soil** (red line) at 4.5K. Relative comparisons show that the slow oxygenation treatment has the most prominent sextet of all. This suggests a larger abundance of more-crystalline Fe(III)-(oxyhydr)oxides that magnetically order in the slow oxygenation treated soil and is consistent with the 140 and 77K data.

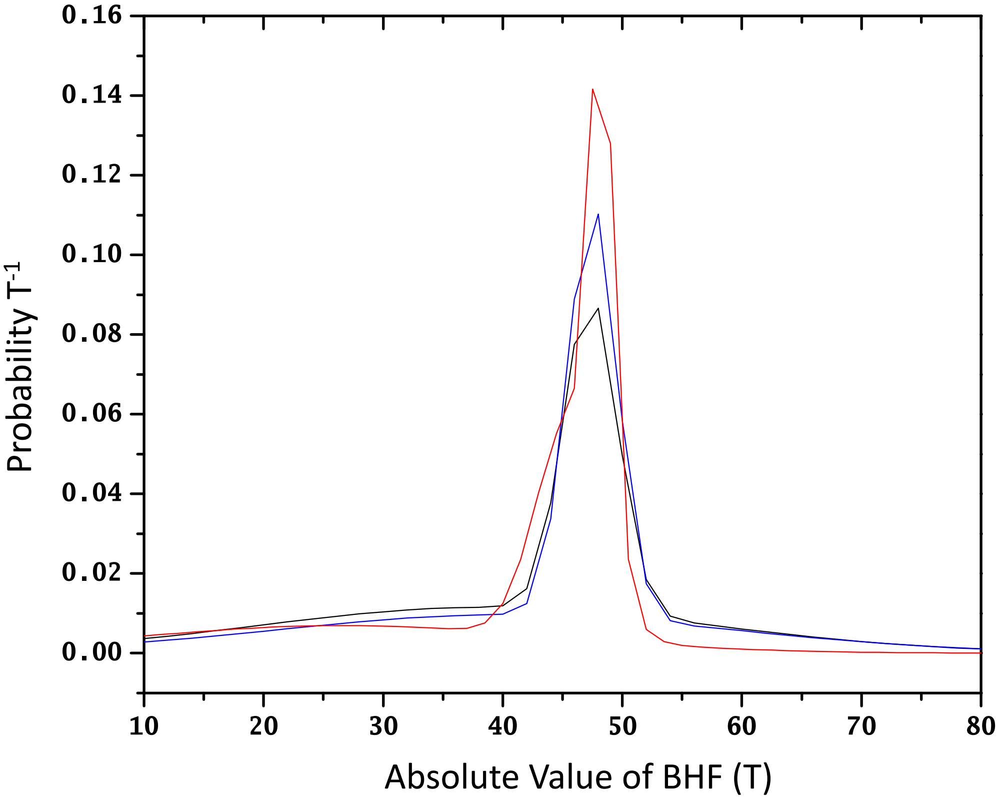

SI Figure S12 Superimposed sextet (HFD-OxHy) Bhf distributions of the air-dried **initial soil** (black line), **fast oxygenation treated soil** (blue line) and **slow oxygenation treated soil** (red line) at 77K. Relative comparisons show that both treated samples have more prominent Bhf probability maxima between 45 T and 50 T compared to that of the initial soil, and the slow oxygenation treatment has the largest Bhf probability maximum of all. This suggests a larger abundance of more-crystalline Fe(III)-(oxyhydr)oxides that magnetically order in the slow oxygenation treated soil, which we attribute to slower oxygenation rates during redox cycling.

Section 3. Additional Fe Characterization

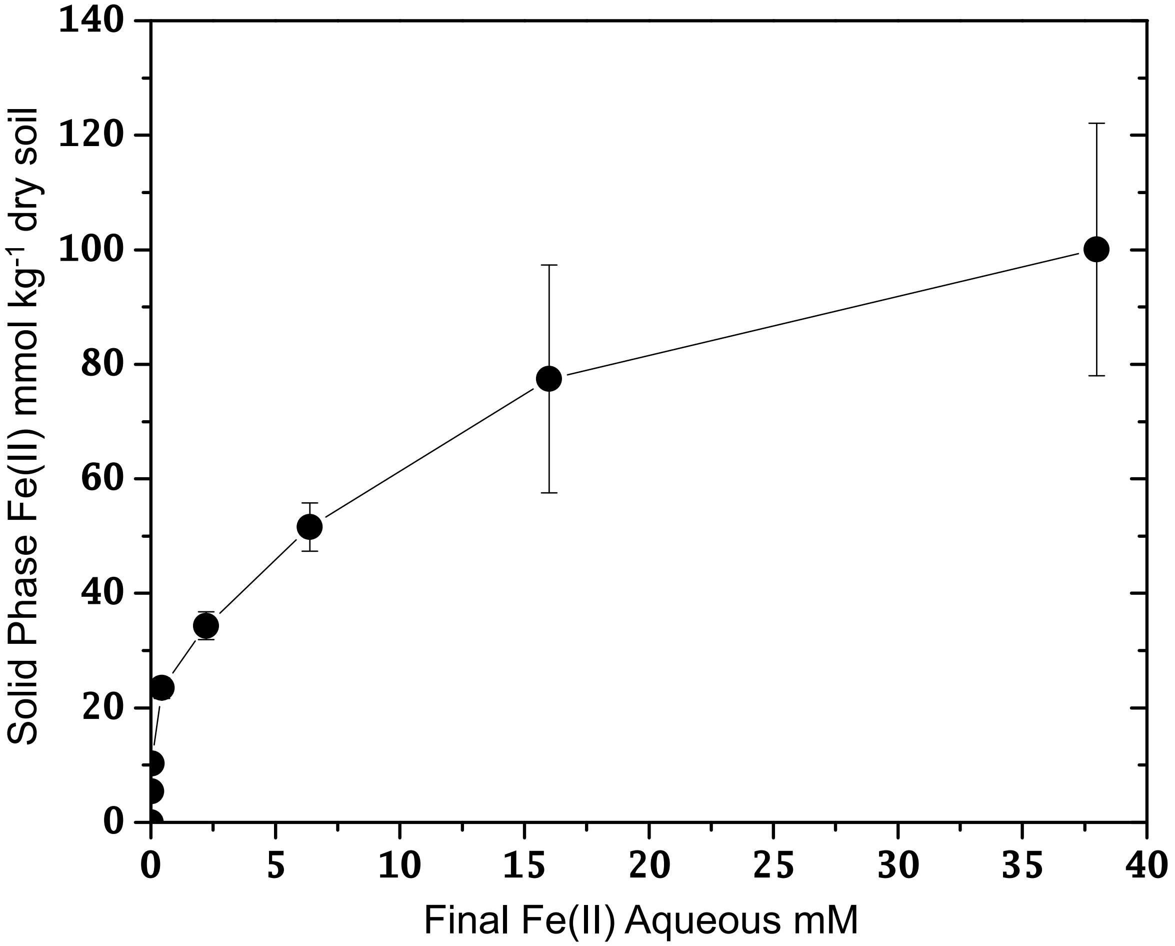

SI Figure S13 Fe(II) sorption isotherm. Soils were mixed in a 10:1 solution:solid ratio for 2 h on a rotary shaker at 200 rpm with 25 mM MES buffer adjusted to final pH 6.0 and final ionic strength of standard solutions was adjusted with KCl to equal that of the 50 mM aqueous Fe(II) standard. The isotherm shows Fe(II) in the solid phase beginning to reach saturation by 30 mM $Fe(II)_{aq}$. This result is consistent with slower net Fe(III) reduction rates as $Fe(II)_{aq}$ reached 30 mM by the end of incubation, indicating that microbial Fe(III) reduction in the solid phase is inhibited by increasing Fe(II) sorption.

References

1. Peretyazhko T, Sposito G. Iron(iii) reduction and phosphorous solubilization in humid tropical forest soils. Geochimica Et Cosmochimica Acta. 2005;69:3643-3652.
2. Hall SJ, McDowell WH, Silver WL. When wet gets wetter: Decoupling of moisture, redox biogeochemistry, and greenhouse gas fluxes in a humid tropical forest soil. Ecosystems. 2013;16:576-589.
3. Thompson A, Chadwick OA, Rancourt DG, Chorover J. Iron-oxide crystallinity increases during soil redox oscillations. Geochimica Et Cosmochimica Acta. 2006;70:1710-1727.
4. Tveit AT, Urich T, Svenning MM. Metatranscriptomic analysis of arctic peat soil microbiota. Applied and Environmental Microbiology. 2014;80:5761-5772.
5. Gifford SM, Sharma S, Rinta-Kanto JM, Moran MA. Quantitative analysis of a deeply sequenced marine microbial metatranscriptome. Isme Journal. 2011;5:461-472.
6. He SM, Wurtzel O, Singh K, Froula JL, Yilmaz S, Tringe SG, Wang Z, Chen F, Lindquist EA, Sorek R, Hugenholtz P. Validation of two ribosomal rna removal methods for microbial metatranscriptomics. Nature Methods. 2010;7:807-U858.
7. Zhang JJ, Kobert K, Flouri T, Stamatakis A. Pear: A fast and accurate illumina paired-end read merger. Bioinformatics. 2014;30:614-620.
8. Buchfink B, Xie C, Huson DH. Fast and sensitive protein alignment using diamond. Nature Methods. 2015;12:59-60.
9. Huson DH, Beier S, Flade I, Gorska A, El-Hadidi M, Mitra S, Ruscheweyh HJ, Tappu R. Megan community edition - interactive exploration and analysis of large-scale microbiome sequencing data. Plos Computational Biology. 2016;12.
10. Ginn BR, Habteselassie MY, Meile C, Thompson A. Effects of sample storage on microbial fe-reduction in tropical rainforest soils. Soil Biology & Biochemistry. 2014;68:44-51.
11. Ginn B, Meile C, Wilmoth J, Tang YZ, Thompson A. Rapid iron reduction rates are stimulated by high-amplitude redox fluctuations in a tropical forest soil. Environmental Science & Technology. 2017;51:3250-3259.
12. Rancourt DG, Ping JY. Voigt-based methods for arbitrary-shape static hyperfine parameter distributions in mossbauer-spectroscopy. Nuclear Instruments & Methods in Physics Research Section B-Beam Interactions with Materials and Atoms. 1991;58:85-97.
13. Rancourt DG, Ping JY. Voigt-based methods for arbitrary-shape static hyperfine parameter distributions in mössbauer-spectroscopy. Nucl Instrum Methods Phys Res, Sect B. 1991;58:85-97.
14. Rancourt DG. Mössbauer spectroscopy in clay science. Hyperfine Interact. 1998;117:3-38.
15. Lalonde AE, Rancourt DG, Ping JY. Accuracy of ferric/ferrous determinations in micas: A comparison of mössbauer spectroscopy and the pratt and wilson wet-chemical methods. Hyperfine Interact. 1998;117:175-204.
16. Kukkadapu RK, Zachara JM, Fredrickson JK, McKinley JP, Kennedy DW, Smith SC, Dong HL. Reductive biotransformation of fe in shale-limestone saprolite containing

fe(iii) oxides and fe(ii)/fe(iii) phyllosilicates. Geochimica Et Cosmochimica Acta. 2006;70:3662-3676.
17. Wu T, Shelobolina E, Xu H, Konishi H, Kukkadapu R, Roden EE. Isolation and microbial reduction of fe(iii) phyllosilicates from subsurface sediments. Environmental Science & Technology. 2012;46:11618-11626.
18. Tishchenko V, Meile C, Scherer MM, Pasakarnis TS, Thompson A. Fe2+ catalyzed iron atom exchange and re-crystallization in a tropical soil. Geochimica Et Cosmochimica Acta. 2015;148:191-202.
19. Morup S, Madsen MB, Franck J, Villadsen J, Koch CJW. A new interpretation of mossbauer-spectra of microcrystalline goethite - super-ferromagnetism or super-spin-glass behavior. Journal of Magnetism and Magnetic Materials. 1983;40:163-174.
20. Madsen MB, Morup S, Koch CJW. Asymmetric doublet in mossbauer-spectra of superparamagnetic goethite. Hyperfine Interactions. 1988;42:1059-1062.
21. Murad E. Clays and clay minerals: What can mössbauer spectroscopy do to help understand them? Hyperfine Interactions. 1998;117:39-70.
22. Murad E, Cashion J: *Mössbauer spectroscopy of environmental materials and their industrial utilization.* Norwell, Massachusetts: Kluwer Academic Publishers Group; 2004.
23. Murad E, Wagner U. Mössbauer study of pure illite and its firing products. Hyperfine Interact. 1994;91:685-688.
24. Rancourt DG, Thibault P-J, Mavrocordatos D, Lamarche G. Hydrous ferric oxide precipitation in the presence of nonmetabolizing bacteria: Constraints on the mechanism of a biotic effect. Geochim Cosmochim Acta. 2005;69:553-577.
25. Thompson A, Rancourt DG, Chadwick OA, Chorover J. Iron solid-phase differentiation along a redox gradient in basaltic soils. Geochimica Et Cosmochimica Acta. 2011;75:119-133.
